# Native Top–Down Analysis of Membrane Protein Complexes Directly From *In Vitro* and Native Membranes

**DOI:** 10.1016/j.mcpro.2025.100993

**Published:** 2025-05-14

**Authors:** Wonhyeuk Jung, Aniruddha Panda, Jaywon Lee, Snehasish Ghosh, Jared B. Shaw, Kallol Gupta

**Affiliations:** 1 Nanobiology Institute, Yale University, West Haven, Connecticut, USA; 2 Department of Cell Biology, Yale University School of Medicine, New Haven, Connecticut, USA; 3 Department of Chemistry, University of Nebraska-Lincoln, Lincoln, Nebraska, USA

**Keywords:** native top–down mass spectrometry, membrane protein complex, electron capture fragmentation, drug binding, proteoform analysis

## Abstract

Macromolecular organization of proteins and lipids in cellular membranes is fundamental to cell functionality. Recent advances in native mass spectrometry (nMS) have established it as a key analytical tool for capturing these associations. This typically necessitates the extraction of target membrane proteins (MPs) from their physiological environments into detergent-like surroundings. In our recent studies using *in vitro* synthetic liposomes, we discovered that gas phase supercharging can selectively destabilize lipid bilayers and enable MS1 detection of embedded and associated protein–lipid complexes. Here, we further extend and apply this methodology to native cell-derived membrane vesicles. We demonstrate our ability to detect and ID protein complexes and their proteoforms directly from native membranes using supercharger-assisted prequadrupole activation followed by downstream native top–down tandem mass spectrometry, which combines both collision-based and electron capture–based fragmentation approaches. We first demonstrated this approach through native top–down identification of several integral MPs from *in vitro* membranes. Subsequently, we developed a protocol to produce nMS-ready native membrane vesicles. Applying to *Escherichia coli* total membranes, we generated nMS-ready vesicles and identified both integral and membrane-associated protein complexes of homomeric and heteromeric nature using our supercharging-enabled native top–down platform. For the heteropentameric β-barrel–assembly machinery (BAM) complex, which includes the integral MP BAM-A, we detected several lipidated proteoforms. For peripheral homodimeric dihydrolipoyl dehydrogenase, we identified bound endogenous metabolite cofactors. Furthermore, using BAM complex, a crucial antibiotic target, we show how this platform could be utilized to study drug binding to MPs directly from their native membranes.

## Highlights

• A supercharger-assisted native top–down analysis platform for membrane proteins (MPs) from *in vitro* and physiological membranes.
• Oligomeric state, proteoforms, and endogenous ligands of MPs and membrane-associated proteins identified without detergent extraction.
• Detection of drug binding of MPs directly from the physiological membrane.

## Graphical Abstract

**Figure:**
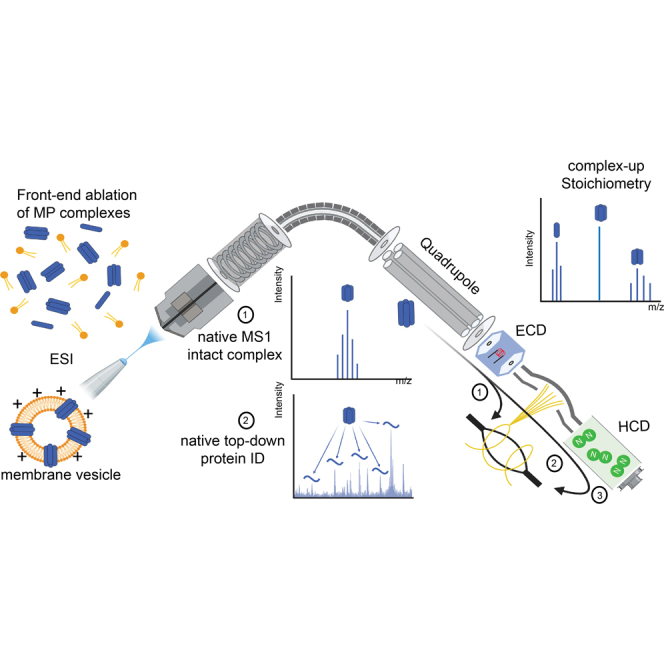

## In Brief

We present a native mass spectrometry platform enabling native top–down analysis of protein complexes from both in vitro and cell-derived membrane vesicles. By integrating homogeneous vesicle preparation, prequadrupole supercharging-assisted activation, and complementary fragmentation methods (CID and ECD), the approach supports detailed characterization of integral and peripheral membrane proteins. Applied to *Escherichia coli* vesicles, it reveals lipidated proteoforms, bound metabolites, and ligand interactions with protein targets. This method provides a powerful tool for studying membrane protein identity, organization, and function in near-physiological settings.

The macromolecular organization of protein in the biological membrane is fundamental to all membrane-associated signaling events (1). The biogenesis of these assemblies is often further regulated by specific physicochemical properties of the biological membrane itself (2, 3, 4). Hence, the most desirable way to study membrane proteins (MPs) is to study them directly from their resident native membrane itself. From determining structures to understanding function, recent trends in technological advancements in all aspects of membrane biology research highlight this pressing need to decipher the workings of MPs by studying them directly in their native membrane context (5, 6, 7). Over the last decade, mass spectrometry (MS) has emerged as a key bioanalytical tool. Early studies of application of MS relied on liquid chromatography–based separation and introduction of target MPs into MS to determine accurate intact monomeric mass and also apply this on crude samples to detect and identify independent proteins (8, 9, 10, 11, 12, 13). Subsequent application of this approach on oligomeric multiprotein complexes revealed the utility in determining accurate masses of different subunits in hetero-oligomeric complexes of MPs (14). These critical technological developments brought us to the next technological question: can we study MP complexes, without denaturing, in their native oligomeric states with bound lipids or ligands? Addressing this, native mass spectrometry (nMS) has emerged as a key technological platform to detect and decipher the organization state of MPs and lipids (15, 16, 17, 18, 19). Its dynamic and ever-expanding mass range (20, 21, 22), high resolution and sensitivity (23, 24, 25), and various tandem mass spectrometry (MS/MS) capabilities provide the most unambiguous molecular resolution to determine the molecular identity and oligomeric stoichiometries of both membrane and soluble protein complexes (15, 26, 27, 28, 29, 30, 31). Nevertheless, a pressing bottleneck of nMS analysis of MP is that it generally demands prior removal of target MP from the biological membranes through amphipathic detergents. Subsequently, the detergent-solubilized purified target MPs are subjected to nMS analysis. This presents a fundamental technological limitation in studying MP complexes directly from physiological membranes using nMS. In typical nMS analysis of detergent encapsulated MPs, the MPs are ablated out of the detergent micelle inside the MS through the use of ergodic collision-induced dissociation (CID). CID provides vibrational excitation to the proteodetergent micelles, leading to their disruption and downstream detection of the liberated MP. However, such ergodic excitation is typically insufficient to dissociate the biological membrane and ablate the integral or peripherally associated proteins (PMPs). This is highlighted in a seminal work that demonstrated that MS-based CID energy alone is insufficient to directly detect MP complexes from intact cellular membranes, and this could only be achieved when membrane vesicles were first disrupted in solution into membrane fragments by sonic excitation and subsequently subjected to CID-based MS activation taking place at the collision cells situated after the quadrupole (32). This reliance on postquadrupole CID to ablate the MPs also imposes a critical limitation to further ID the ablated protein through quadrupole isolation and top–down fragmentation, restricting nMS to MS1-only intact mass analysis. However, in the native membrane context, mass alone is mostly insufficient to unambiguously determine the identity of the detected protein. Recent work has further shown the use of other MS-based energies, such as infrared-based activation (26). Nevertheless, the energy framework available inside MS, either using collisional or infrared-based activation, is typically insufficient to complete the dispersion of the physiological membrane. Consequently, successful application of the approach that relies on CID or other MS-based excitation to dislodge MPs from the native membrane has remained limited to only specialized membranes that have unusually high protein-to-lipid ratio and are naturally enriched with only one MP, like the purple membranes or retinal membranes (18, 33, 34). Together, these seminal works both raise the prospect of studying an MP from its native context and simultaneously pose a technical question: how can we supplement available MS-activation methods to directly ablate and study MPs directly for variety of native physiological membrane environments.

We recently discovered that lipid bilayers can be weakened exclusively in the gas phase during the electrospray process with the help of a small amount of supercharger added to the spray solution (35). Using this, we showed that MP–lipid complexes can be detected directly from intact proteoliposomes to determine functional organization and lipid-binding specificity of MPs, directly from tunable *in vitro* bilayer (35, 36, 37). However, this previous work exclusively relied on MS1-only intact mass analysis of purified known MPs from reconstituted bilayers. In this work, we extend this method to develop a technological platform to detect and identify native endogenous protein complexes directly from the native cell membrane vesicles through native top–down (nTD) analysis. To achieve this, we further coupled this with electron-capture dissociation (ECD)–based nTD fragmentation to show that we can directly detect homomultimeric and heteromultimeric complexes, unambiguously identify them through top–down fragmentation, and can also use this platform to study drug–ligand binding to proteins directly from the membrane. The use of nonergodic ECD fragmentation allows the fragmentation of large complexes and their proteoforms, overcoming the size limitation of CID because of its ergodic nature (38, 39, 40, 41, 42, 43, 44, 45). This is specifically highlighted for MPs in a series of seminal works, where in combination with LC-based fractionation, nonergodic electron-based fragmentations revealed orthogonal information to collision-based fragmentation approaches, revealing critical information on subunit composition and increased hydrophobic transmembrane domain coverage (13, 46). Consequently, we incorporated ECD within our experimental framework to demonstrate an attempt to apply the methodology to intact native membrane vesicles. Taking gram-negative bacterial membrane as a model system, we first develop a sample preparation strategy that delivers nMS-ready intact native membrane vesicles. Subsequently, we demonstrate the results of applying the lipid vesicle nTD strategy to intact physiological membrane vesicles to identify multiprotein complexes and their proteoforms, bound lipids, and endogenously and exogenously added ligands.

## Experimental Procedures

### Recombinant Protein Expression and Purification

All recombinant proteins were purified using previously published protocol (15, 35, 47, 48, 49, 50). All details can be found in the Supplemental Methods section.

#### Preparation of nMS–Ready Proteoliposomes

nMS-ready proteoliposomes were prepared following the previously described protocol (35, 51). Briefly, Sephadex G-50 powder in 200 mM ammonium acetate (NH_4_Ac) was sonicated in a water bath for 5 min and then was left to swell overnight while being degassed under a vacuum. Then, an empty column was packed with the swollen Sephadex. Concomitantly, *Escherichia coli* Polar Lipid Extract (Avanti Polar Lipids) was dried to form a lipid film and then resuspended in 200 mM NH_4_Ac and 2 mM DTT. Then, the solution was sonicated in a water bath for 15 min, and 10 freeze–thaw cycles were performed (liquid nitrogen (N_2_) to 50  °C water bath) to form liposomes. Afterward, detergents were treated to reach the final concentration of 2X critical micelle concentration. After a 30-min on-ice incubation, respective proteins in detergents were added to the liposomes to achieve lipid: protein ratios ranging from 100 to 1000. The samples were incubated on ice for 2 h and then placed atop a set-up column and underwent gel filtration to isolate the proteoliposome fraction. Finally, a quality check of the proteoliposomes was performed with electron microscopy and dynamic light scattering (DLS) (DynaPro Nanostar; Wyatt Technology).

### Preparation of nMS-Ready Bacterial Membrane Vesicles

*E. coli* strain BL21 (Sigma–Aldrich; CMC0020) (52, 53) was cultured in 1 l LB overnight. The cells were pelleted and then resuspended in 50 ml lysis buffer (20 mM Tris, 150 mM NaCl, pH 7.4) supplemented with protease inhibitor cocktail tablets (see aforementioned) and DNase I (Roche Diagnostics) at 10 μg/ml, and DTT at 2 mM and then lysed using a cell disruptor (Avestin). Cell debris was removed by centrifugation (20,000*g*, 20 min, 4 °C). From the supernatant, the membranes were pelleted by ultracentrifugation (200,000*g*, 4 h, 4 °C). The membrane was resuspended with 15 ml of ice-cold 500 mM NH_4_Ac. The resuspended membrane was homogenized and then subjected to cavitation with a Cell Disruption Vessel (Parr Instrument Company). After loading the vessel with the homogenized membrane, N_2_ was fed into the chamber until approximately 750 psi was reached and then the chamber was incubated in ice for 20 min. DLS analysis (DynaPro Nanostar) was performed to confirm uniformity and optimal size (100–200 nm diameter) for subsequent nMS-based analysis. The sample was further cleaned up with Pierce Protein Concentrator PES, 100 K molecular weight cutoff (Thermo Fisher Scientific) and then bicinchoninic acid assay (BCA assay) was performed with the Pierce BCA Protein Assay Kits to ensure that the final protein concentration of the sample was approximately 300 μg/ml.

### Transmission Electron Microscopy Analysis of nMS-Ready Bacterial Membrane Vesicles

The morphology and size of the bacterial membrane vesicles before and after N_2_ cavitation were investigated by negative stain transmission electron microscopy. The samples were prepared by diluting 1:10 and 1:100, with 200 mM NH_4_Ac solution. Carbon-coated copper grids (200 mesh) were glow-discharged for 30 s. Five microliters of sample was applied to the grid and after 60 s blotted off with Whatman (ashless) filter paper and subsequently washed once, with uranyl formate (2% in H_2_O), and then finally stained with uranyl formate for 60 s and blotting dry using Whatman filter paper. Micrographs were taken on a JEOL JEM 1400PLUS electron microscope using an operating voltage of 80 kV. The average size of the membrane vesicles was estimated from at least 30 or more well-defined individual particles.

### MS Analysis

#### nMS and nTD-MS Analysis of Proteoliposomes in Q-Exactive UHMR

The nMS analysis of the proteoliposomes and membrane vesicles was performed as previously described (35). The protein concentration was kept between 2 μM and 10 μM for optimal nMS analysis. The sample was then subjected to nanoelectrospray ionization with pulled nanoemitter capillaries equipped with platinum wire (see supplemental data for details). All nMS and nTD-MS data were acquired by Q Exactive UHMR (Thermo Fisher Scientific), which was modified with ExD TQ-160 cell (e-MSion; Agilent, see later for more details). Thermo Tune software (Q Exactive UHMR 2.13 Build 3159) was used to control the instrument, and the data were analyzed using FreeStyle 1.7 (MS1 & MS2; Thermo Fisher Scientific), ExDViewer, version 4.5.6 (MS2; e-MSion, Agilent), ProSight Native v1.0.24103 (MS1 & MS2; Proteinaceous), and ProSight Lite online (MS2; Proteinaceous). Annotation results from ExDViewer were manually verified, exported, and then further modified *via* Adobe Illustrator to generate all annotation figures. The key parameters for the MS1 analysis of the buffer-exchanged VAMP2, semiSWEET, and AqpZ were spray voltage 0.8 to 1.2 kV, resolution 6250, capillary temperature 300 °C, desolvation voltage −225 V to −75 V. The nMS-ready-proteoliposomes were spiked with a supercharging agent, 3-nitrobenzyl alcohol to the final concentration 0.1% (v/v) to facilitate MP ejection from the liposomes. Electron capture higher-energy collisional dissociation (EChcD) (52, 53) was applied to induce fragmentation. The key parameters for the MS2 analysis of the proteoliposomes are as follows: spray voltage 0.8 to 1.2 kV, resolution 100 k (200 k for AqpZ), higher-energy collisional dissociation (HCD) energy 80 to 250 V, capillary temperature 300 °C, desolvation voltage −150 to −120 V, RF amplitudes of injection flatpole 700, bent flatpole 940, transfer multipole 600, C-trap 1600, and trapping gas 1 to 2. The transfer multipole of Q-Exactive UHMR was replaced with an ExD cell. For each MS spectra, 2000 to 3000 microscans were averaged to a single scan. The resulting final MS2 data were first analyzed with ProSight Native with the database that includes the total *E. coli* proteome and VAMP2. Then, ExDViewer was used to manually verify each fragment and calculate sequence coverage.

#### nMS, nTD-MS, and Native Complex Up Analysis of Proteins from *E. coli* Membrane Vesicles in Q-Exactive UHMR

*E. coli* strain BL21 membrane vesicles solubilized in 500 mM NH_4_Ac were diluted to the final concentration of 300 μg/ml and spiked with glycerol to the final concentration of 5% (v/v) to induce supercharging and facilitate MP ejection. The nMS parameters were optimized to detect as many species as possible while maintaining enough intensity for subsequent nTD-MS and native complex up (nCU) analysis for the major species detected. The sample was introduced to the instrument using in-house pulled nanospray emitters. The pulling parameters were optimized to obtain an aperture diameter between 4 and 5 μm and a taper angle of 10° to 15°. The key parameters for nMS analysis were spray voltage 1.2 to 1.5 kV, resolution 3125, HCD energy 5 V, capillary temperature 300 °C, and desolvation voltage −160 V.

The key parameters for the nTD experiment were spray voltage 1.2 kV, resolution 100 k, HCD energy 250 V, capillary temperature 250 °C, desolvation voltage −100, RF amplitudes of injection flatpole 700, bent flatpole 940, transfer multipole 600, C-trap 1600, and trapping gas 2. The ExD cell voltage profile was optimized to obtain the best fragmentation spectra. For each MS/MS data, ∼2000 microscans were averaged to a single scan. The MS2 data were then separately deconvoluted using FLASHDeconv (48) and ExDViewer. The deconvoluted data were then used for protein identification using ProSight Native v1.0.24103 (54) (Proteinaceous). For the nCU experiment to examine the oligomeric state and the bound cofactor. ExD cell was turned off, and only HCD was applied until robust monomer ejection could be detected.

The β-barrel–assembly machinery (BAM) complex was partially denatured by treating the membrane vesicles with NH_4_OH to the final concentration of 2% (v/v) before nTD-MS analysis. The 19+ charge state of the 99,610 Da species was isolated and then subjected to nTD-MS analysis *via* EChcD following the above-described protocol.

The BAM A inhibitor MRL-494 was purchased from MedChemExpress (catalog no.: HY-128773) and was treated to the membrane vesicle to the final concentration of 100 μM. After 1 h incubation on ice, the mass spectrum was acquired. The MRL-494-bound BAM complex was isolated and subjected to nCU analysis by applying HCD 200V.

## Results

We broke down the task of nTD analysis from physiological membranes into three major subgoals. First, using *in vitro* lipid vesicles, we developed an experimental strategy that enables gentle prequadrupole ablation of protein complexes from the membrane for their MS1 detection, subsequent quadrupole mass selection of target complexes, and final, downstream nTD to identify the complex. Next, we developed a sample preparation strategy that delivers nMS-ready homogeneous intact membranes from the physiological membranes. Finally, we show an application of this strategy on gram-negative bacterial membranes.

### Establishing a Platform for nTD Analysis of MPs Directly from the Membrane

**Fig. 1:**
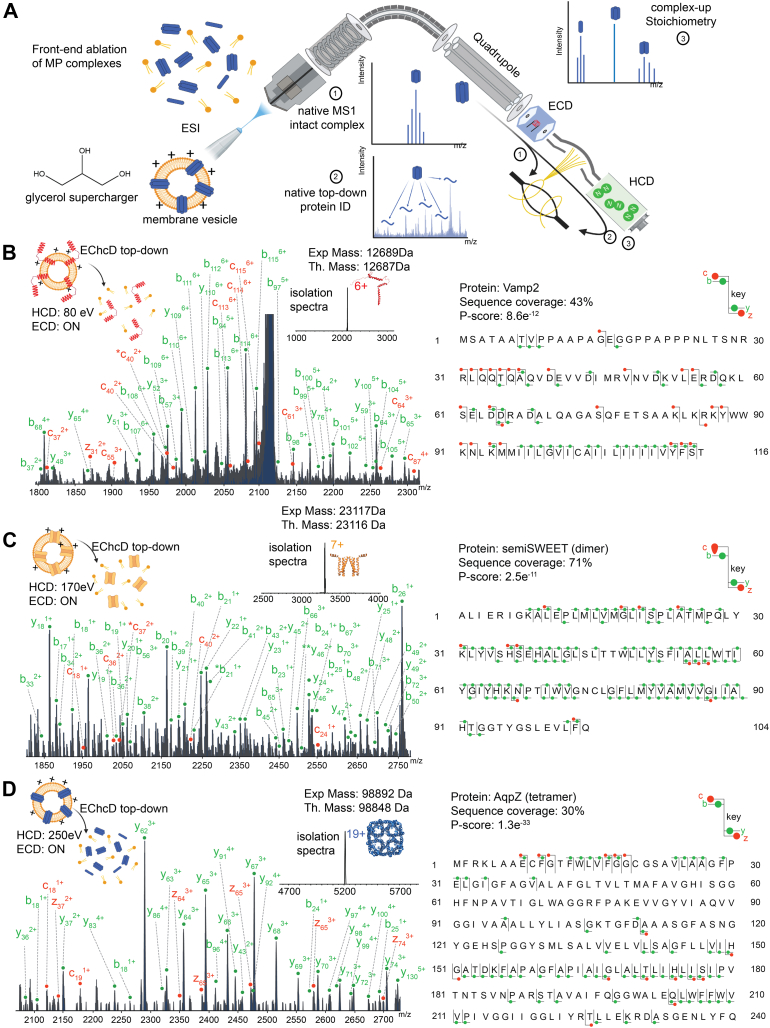
**Native top–down mass spectrometry (nTD-MS) analysis of membrane proteins (MPs) from *in vitro* liposomes.***A*, schematic representation of MP analysis from membrane vesicle *via* nMS. MPs are ablated out of the lipid bilayer by utilizing the front-end activation and are directly detected (1). This is facilitated by supercharging agents such as glycerol, which increases the amount of charge deposited onto the vesicle during the ESI process. The liberated MP complexes are quadrupole isolated for downstream native complex-up (nCU) experiment, where the complex is dissociated into its components (2). Alternatively, the isolated species can be subjected to nTD-MS analysis in which they undergo fragmentation by CID or EChcD (3). Proteoliposomes formed by incorporating VAMP2 (*B*), semiSWEET (*C*), and AqpZ (*D*) were subjected to nTD analysis. The physiological oligomeric states of the respective proteins (VAMP2 [monomer], semiSWEET [dimer], and AqpZ [tetramer]) were quadrupole isolated and fragmented with EChcD approach, and a representative section of the corresponding spectra is shown. The MS2 data were searched against the modified database that includes the total *Escherichia coli* proteome supplemented with VAMP2, semiSWEET, and AqpZ sequences. The respective *P* scores are displayed here along with the sequence coverage. The b/y and c/z fragments are highlighted in *green* and *red*, respectively. The experimental and theoretical masses of the proteins, in their physiological oligomeric states, are denoted as Exp and Th. Mass, respectively. The HCD voltage and the status of the ECD cell are stated in each MS/MS spectrum. CID, collision-induced dissociation; ECD, electron-capture dissociation; EChcD, electron capture higher-energy collisional dissociation; ESI, electrospray ionization; HCD, higher-energy collisional dissociation; nMS, native mass spectrometry.

We first demonstrate our ability to identify and determine the stoichiometric organization of protein complexes, directly from the lipid vesicles, through nTD analysis. Our goal was to set up an experimental pipeline to eject proteins from the membrane vesicles through in-source activation, select specific protein complexes through quadrupole isolation, and perform nTD analysis of the selected species. Here, owing to the larger molecular weights and lower charge states of native MP complexes, we reasoned that a general platform should include fragmentation approaches beyond CID. This is because the ergodic nature of commonly used CID fragmentation poses a limitation on the efficient fragmentation of large native protein complexes, as demonstrated through nonergodic fragmentation-based TD experiments on both soluble and detergent-encapsulated MPs (39, 55, 56, 57, 58, 59, 60, 61, 62, 63, 64). To achieve this, we decided to supplement CID with nonergodic ECD fragmentation and retrofitted our instrument with a commercially available ExD cell using previously established protocols (29, 53, 65, 66). The ExD cell is fitted between the quadrupole and the collision cells, replacing the transfer multipole. This provides an experimental avenue to ablate MP complexes from the membrane in the source region and quadrupole isolate a specific protein complex species of interest (Fig. 1A). The isolated ions then capture electrons on their way to the C-trap and are subsequently subjected to further collisional activation in the collision cells to provide a hybrid fragmentation termed EChcD (67, 68). Finally, high-resolution MS/MS spectra of the generated fragment ions are obtained in the orbitrap (Fig. 1A). Before applying this to native membranes, we set up this experimental platform for nTD experiments on MPs from the membranes through targeted analysis of specific model MPs from *in vitro* lipid vesicles.

**Fig. 2:**
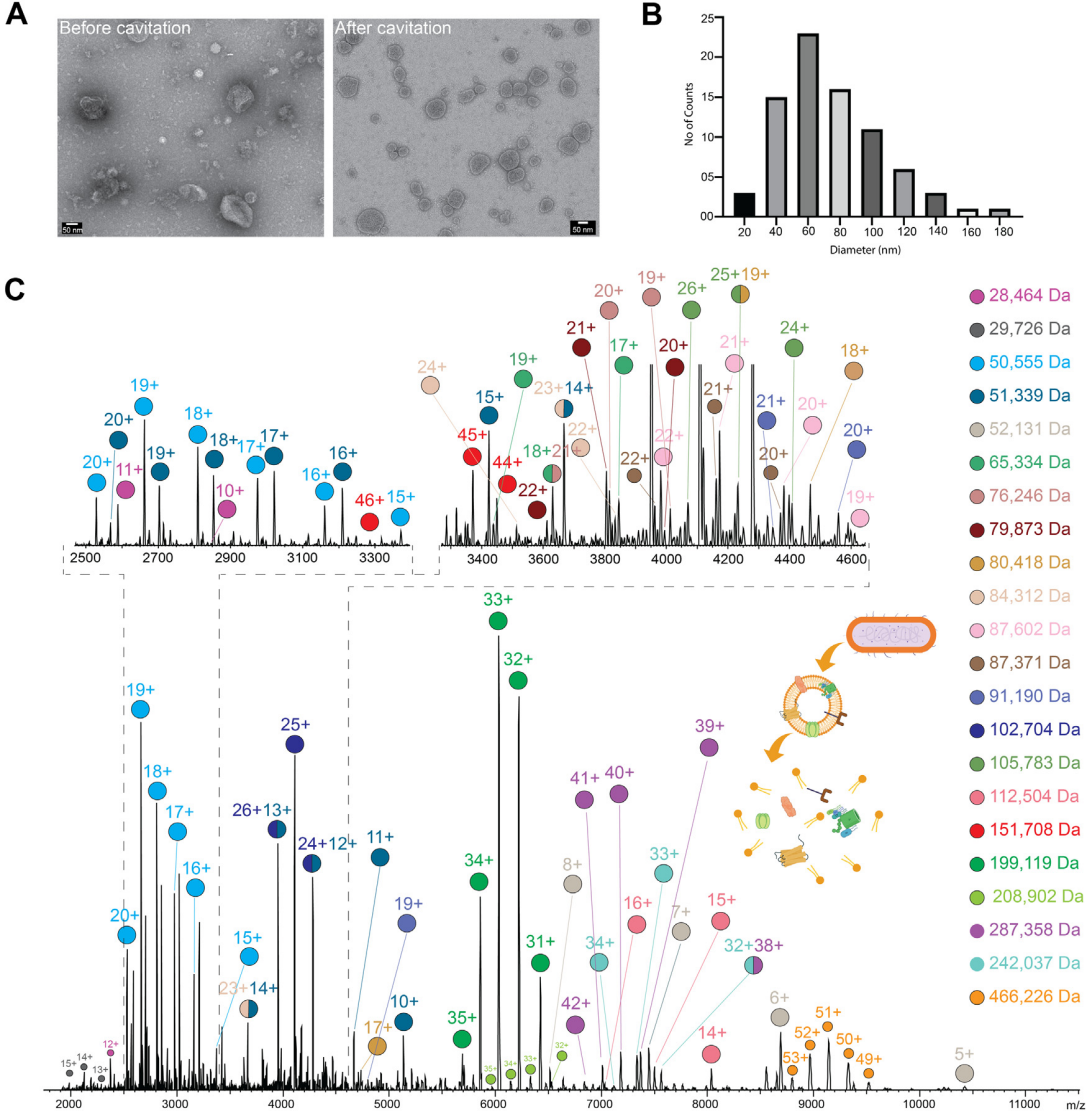
**Proteins ejected out of membrane vesicles analyzed *via* nMS.***A*, EM picture of *Escherichia coli* membrane vesicles before and after N2 cavitation. *B*, nMS analysis of the cavitated vesicles yields a variety of endogenous proteins/protein complexes ranging from 28 to 466 kDa. nMS, native mass spectrometry. *C*, the size distribution of the vesicles was measured by manual counting. The vesicles are relatively homogeneous, with 68% of the vesicle’s diameter ranging from 40 to 80 nm, confirming that cavitation generates small vesicles that are homogeneous in size.

To examine the general applicability of this approach, we picked three different integral MPs (IMPs), VAMP2, semiSWEET, and AqpZ. Their oligomeric states range from monomer to dimer to tetramer, and their mass ranges from 12 to 100 kDa. Each of these proteins was independently reconstituted into proteoliposomes mimicking the lipid compositions of native *E. coli* membranes. Subsequent nMS analysis of the proteoliposomes yielded successful detection of the native oligomeric states of the respective MPs directly from the liposome using in-source activation (supplemental Fig. S1). Subsequently, specific charge states of each of these MPs were directly isolated and subjected to EChcD analysis, where each isolated precursor was first subjected to electron capture followed by collisional activation in the collision cell (Fig. 1 and supplemental Fig. S2).

While for VAMP2, monomer charge states were selected for nTD, for semiSWEET and AqpZ, dimeric and tetrameric charge states were respectively subjected to nTD analysis. In the native membrane, we expect the bulk of the proteins to remain in physiological oligomeric states. Accordingly, while picking monomeric charge states would have yielded higher sequence coverage, to better mimic the physiological scenario, we restricted our analysis to only the physiological oligomeric states of the respective IMPs (VAMP2-monomer, semiSWEET-dimer, and AqpZ-tetramer) to nTD analysis. In each of these cases, the respective precursor ions were first subjected to ECD, followed by collisional activation. The exact ECD and CID parameters used are tabulated in supplemental Table S1. Only terminal ion types were considered for the current analysis for all three proteins. As shown, for each of these proteins, abundant fragmentation was observed for each of the proteins (Fig. 1, *B*–*D* and supplemental Figs. S2–S4). To mimic a realistic scenario of an unknown protein ID, we first tested the quality of the data in identifying the proteins. To do this, we manually incorporated VAMP2, semiSWEET, and AqpZ sequences into the *E. coli* proteome database to demonstrate the ability to identify the respective MPs with our nTD workflow. The respective MS/MS data were then searched against the modified *E. coli* database using ProsightNative (54). In all these cases, the target protein was identified with very low P-scores (VAMP2: 8.6e^−12^, semiSWEET: 2.5e^−11^, and AqpZ: 1.3e^−33^) from the database. The data were further validated by manual verification of the matched terminal fragment ions. As shown, in each of the three cases, ions matched to the target sequence yielded a significant sequence coverage (VAMP2: 43%, semiSWEET: 71%, and AqpZ: 30%). An important point to note is that for all the proteins, fragment ions could be observed for both cytosolic as well as transmembrane domains. Together, these experiments on *in vitro* membranes established an experimental platform to *de novo* identify MPs through nTD analysis of their physiological oligomeric states, directly from the lipid membrane. Next, we applied this to native membranes.

### Generating nMS-Ready Intact Membrane Vesicles

We first optimized an experimental avenue that can deliver nMS-ready membrane vesicles, directly from intact native membranes. Here, we took inspiration from our previous success in detecting MPs directly from customizable *in vitro* synthetic liposomes (35, 36). We observed that homogeneity, size (up to 300 nm), and unilamellarity of proteoliposomes play critical roles in successfully detecting embedded MP complexes. Hence, we naturally reasoned that they would also be critical factors while attempting to fly cellular membrane vesicles. While there are many ways of generating membrane vesicles, after experimenting with various avenues, we found N_2_ cavitation to yield the most homogeneous nMS-ready membrane vesicles (69). Most critically, we observed that by regulating the N_2_ pressure in the cavitation chambers, we could regulate the vesicle population to remain homogenous and unilamellar between 100 and 200 nm diameter (Fig. 2A); a sweet spot for vesicle sizes and characteristic that we have already determined through our liposome-based work. Furthermore, during N_2_ cavitation, the pressure is evenly distributed throughout the membrane resuspension, preventing local heating (70). This offers a major advantage over local cavitation approaches of membrane perturbation, such as sonication, which can lead to local heating and aggregation. Taking gram-negative bacteria (*E. coli* membrane), the optimal N_2_ cavitation pressure was found to be approximately 750 psi, at which point homogenous membrane vesicles that measure at 100 nm diameter on average are generated. This was confirmed through both DLS and negative-stain imaging (Fig. 2, *A* and *B*).

### Detection of Protein Complexes From the Native Membrane Vesicles

We next proceeded to subject these vesicles to nMS. Here, we leveraged our prior discovery that mild gas phase supercharging can lead to destabilization of the bilayer through selective protonation of the membrane lipids. Our inspiration came from the field of lipid vesicle–based drug delivery, where protonatable lipids are embedded in the membrane to destabilize it from within through selective protonation at the acidic endolysosomal systems (71). We used supercharging agents to emulate this chemistry in the gas phase (72, 73). This is further supported by prior gas phase calculation confirming that several abundant biological lipids, such as phosphatidylcholine and sphingomyelin, have gas phase basicity comparable to that of Arg or Lys residues in the protein (74). However, several key differences between the synthetic membrane and native vesicles prompted us to develop a strategy targeted to the native membrane. We reasoned that a significantly lower lipid:protein ratio of the physiological membranes (between 30:1 and 50:1) compared with the proteoliposomes (1000:1) would demand much softer supercharging conditions. Further, such high-density packing of the physiological membranes often makes the corresponding membrane vesicles aggregation prone, posing a significant challenge for long and stable spray required for nTD MS experiments. Here, while experimenting with a molecular additive that is typically used to increase the membrane stability of cell-derived membrane vesicles, we serendipitously observed that glycerol can serve a dual purpose. While the role of glycerol as a membrane stabilizer is well established (75), we observed that its’ previously reported mild supercharging effect is sufficient to increase the efficiency of ablation of MPs directly from the native membrane (76). Figure 2*C* shows nMS spectra obtained by spraying *E. coli* intact membrane vesicles with 5% glycerol. A point to note is that in our previous work with synthetic membranes, we used much stronger supercharging agents, such as glycerol carbonate or 3-nitrobenzoic acid. We reasoned this is because of the much higher lipid:protein ratio in typical proteoliposomes over physiological membranes (∼20-fold higher lipids/per copy of protein). The higher lipid ratio required a higher degree of supercharging. An independent proof of this can be observed in recent work where nMS was elegantly used to detect oligomers of bacteriorhodopsin directly from the purple membrane (18). Purple membrane is a unique physiological membrane that has an exceptionally low lipid:protein ratio (∼3:1). Hence, rhodopsin could be detected even without using any supercharger but by just using collisional activation.

The nMS spectra of MP ablated from *E. coli* membrane vesicles show the presence of molecular species from 16 kDa to up to 500 kDa (Fig. 2*C*). Further, the enhanced stability of the membrane vesicles because of glycerol enhances spray stability, enables the sample to be continuously sprayed over 2 hours (supplemental Fig. S5). Further, the addition of glycerol enables us to obtain the spectra without using any additional collisional activation at the collision cell, only using in-source activation. As shown in supplemental Fig. S6, removing glycerol, under the same MS conditions, led to a significant loss in the number of detected protein species. This in-source ablation of proteins from the native membrane is an essential requirement for their downstream identification through nTD analysis. This marks a critical technological advancement of this current work, as all prior work required in-source activation as well as further collisional activation in the collision cell to detect MPs, precluding top–down identification of the detected complexes. Our sample preparation protocol, which delivers uniform nMS-ready intact membrane vesicles, coupled with supercharging-aided in-source activation paves a pathway for downstream unambiguous identification through nTD fragmentation. We will demonstrate this in the next section. A key point here is to note that for any given supercharger, the degree of supercharging is also regulated by the size of the starting droplet, which is expected to be between 1/14 and 1/20 of the spray needle diameter (77). Scanning electron microscopy was used to determine our average needle diameter as 4.5 μm (supplemental Fig. S6). We have provided detailed scanning electron microscopy images of the needles used along with the needle-pulling recipe to aid transparency and reproducibility. The needles were also not gold coated, but the electrospray high voltage was applied *via* a platinum wire inserted into the electrospray needle. Needles with different diameters are expected to provide different outcomes and the positioning of the needle relative to the capillary along with the spray voltage will all contribute to the extent of the supercharging observed.

### Identifying Native MP Complexes Directly From the Native Membrane

We next proceeded to perform nTD directly on the native membrane to identify the MS1-detected protein complexes. Proteins associated with biological membranes can be classified as either IMPs, which have transmembrane domains that keep them embedded within the bilayer, or PMPs, which are associated with the membrane through lipidation (like the Ras-proteins) or their noncovalent interactions with the membrane or other MPs (7). Based on their oligomeric organization, these proteins can further be classified as homomeric and heteromeric. Complete identification of native protein complexes requires both determining their identity through nTD, as well as determining their stoichiometry through complex-up analysis, that is, collisional activation for intact complexes into subcomplexes. Taking two specific examples, we show how our approach fares for all these classes of membrane-associated proteins.

**Fig. 3:**
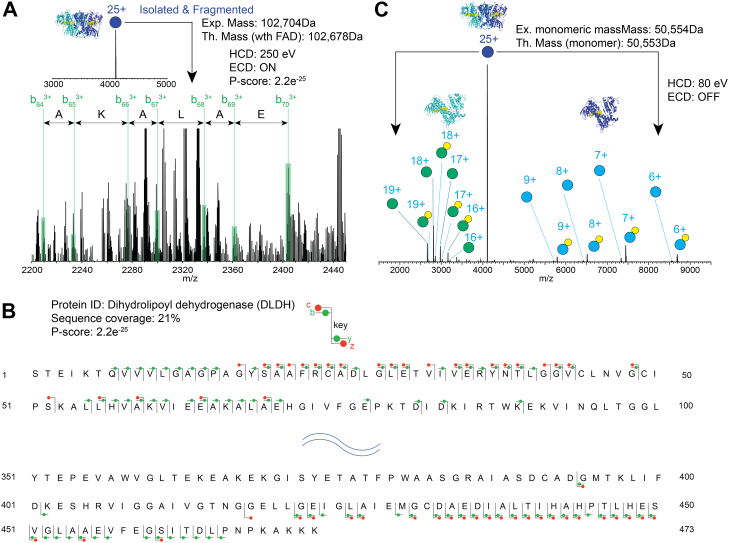
**nTD-MS and complex-up analysis of an endogenous protein from *Escherichia coli* membrane vesicle.***A*, the 25+ charge state of the 102 kDa species was isolated *via* quadrupole and then subjected to nTD-MS analysis with EChcD approach. A representative part of the fragmentation spectra is shown here, and the full annotation is available in Figure 4. *B*, the MS2 data from (*A*) were searched against the *E. coli* membrane proteome database using ProSight Native. As shown, this led to an unambiguous ID of the protein as DLDH with a very confident *P* score, *E* value, and >20% sequence coverage. The annotated terminal ion types are marked on the sequence. *C*, the dimeric endogenous oligomeric state of the protein was further confirmed by subjecting the same precursor to a complex-up analysis by CID. Data show clear ejection of a high charge state unfolded monomers and a low charge state folded monomer, with the endogenous cofactor (FAD, represented in *yellow*) still bound. The experimental and theoretical masses of the FAD-bound dimers are denoted as Exp and Th. Mass, respectively. The HCD voltage and the status of the ECD cell are stated in each MS/MS spectrum. FAD, flavin adenine dinucleotide. CID, collision-induced dissociation; DLDH, dihydrolipoyl dehydrogenase; ECD, electron-capture dissociation; EChcD, electron capture higher-energy collisional dissociation; HCD, higher-energy collisional dissociation; nTD-MS, native top–down mass spectrometry.

**Fig. 4:**
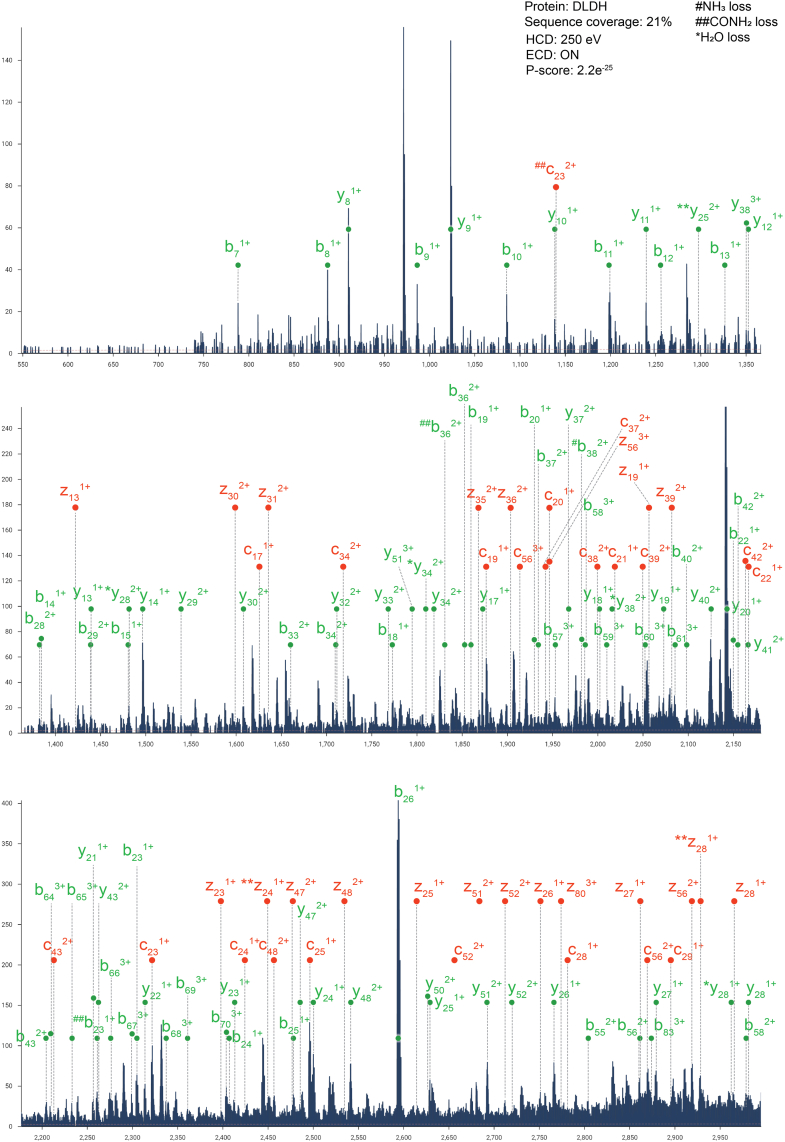
**Annotated nTD-MS data of dimeric DLDH.** 25+ charge state of the dimeric DLDH is quadrupole isolated, subjected to EChcD fragmentation. Sections of annotated EChcD nTD-MS data are shown. Only terminal ions are annotated. Three different neutral losses, NH_3_ (denoted with #), CONH_2_ (denoted with ##), and H_2_O (denoted as ∗), were included. Overall, the data yield 21% sequence coverage. The HCD voltage and the status of the ECD cell are stated in each MS/MS spectrum. DLDH, dihydrolipoyl dehydrogenase; ECD, electron-capture dissociation; EChcD, electron capture higher-energy collisional dissociation; HCD, higher-energy collisional dissociation; nTD-MS, native top–down mass spectrometry.

We first focus on the species with a mass of 102.7 kDa, one of the most intense species detected in the MS1 spectrum (Fig. 2*C*). EChcD-based nTD analysis of the 25+ charge state yielded a robust fragmentation spectrum, with several peaks showing clear isotopic distribution (Figs. 3 and 4). Querying the data against the *E. coli* database using ProsightNative provided an unambiguous identification of the protein as dihydrolipoyl dehydrogenase (DLDH, gene name: lpdA), a PMP (78, 79), with very confident P and E-scores (see Experimental Procedures section for more details on search parameters). Figure 3*B* shows the sequence coverage, with the fragment ion types marked. Further extensive annotations are provided in Figure 4. As shown, a continuous series of N and C-terminal fragment ions were observed yielding >20% sequence coverage. Since the spectra were taken under EChcD conditions, both b/y and c/z ion types were observed. Interestingly, the determined parent mass (102.7 kDa) is a bit more than twice the monomeric mass of DLDH, indicating the protein is probably present in an oligomeric state with some endogenous ligands bound to it. To determine this, we performed a complex-up analysis of the same species. The spectra confirmed the endogenous oligomeric species as a dimer, as a clear ejection of monomer matching the theoretical monomer mass could be detected (Fig. 3*C*). Interestingly, the monomers ejected from the complex were also observed with an additional mass of 785 Da, which corresponds to binding of an endogenous ligand. Searching this mass against the small molecule database (*via* ProsightNative) and filtering them against known small-molecule interactors (*via* UniProt) identified the ligand as flavin adenine dinucleotide, an endogenous cofactor of DLDH that acts as an oxidizing catalyst (78). Together, employing combined nTD and complex-up analysis directly on the physiological membrane, we could determine the identity of a peripheral MP, along with its physiological oligomeric state and bound endogenous ligand. To date, this is the first example of nTD-based identification of an endogenous oligomeric protein complex directly from the intact physiological membranes, without using any detergent or other membrane mimetics. We then focus on nTD-based identifications of a few other proteins that are present in the MS1 spectra of native membrane vesicles. We have successfully identified RbsB (supplemental Fig. S7), superoxide dismutase [Fe] (supplemental Fig. S8), tryptophanase (supplemental Fig. S9), and monomeric DLDH (supplemental Fig. S10). The ability to detect endogenously bound ligands in DLDH presents an exciting avenue to use to study drug binding to MPs by directly studying physiological membranes using nMS. This alleviates the typical need to dislodge the target MP from the membrane to a detergent environment, before performing ligand binding. Indeed, such strategies often present major limitations as both partitioning and diffusion of a candidate drug molecule into the native lipid bilayer membrane usually play a critical role in determining its physiological binding efficacy to the target MP. These phenomena cannot be recapitulated in detergents, often leading to artifactual detergent–based observations of ligand binding to MPs that are not reciprocated in the native membrane, the intended place of action of the drug. Further, studying ligand binding to a target MP, directly from its physiological membrane represents the competitive molecular environment that an actual drug would experience, better representing the physiological scenario.

**Fig. 5:**
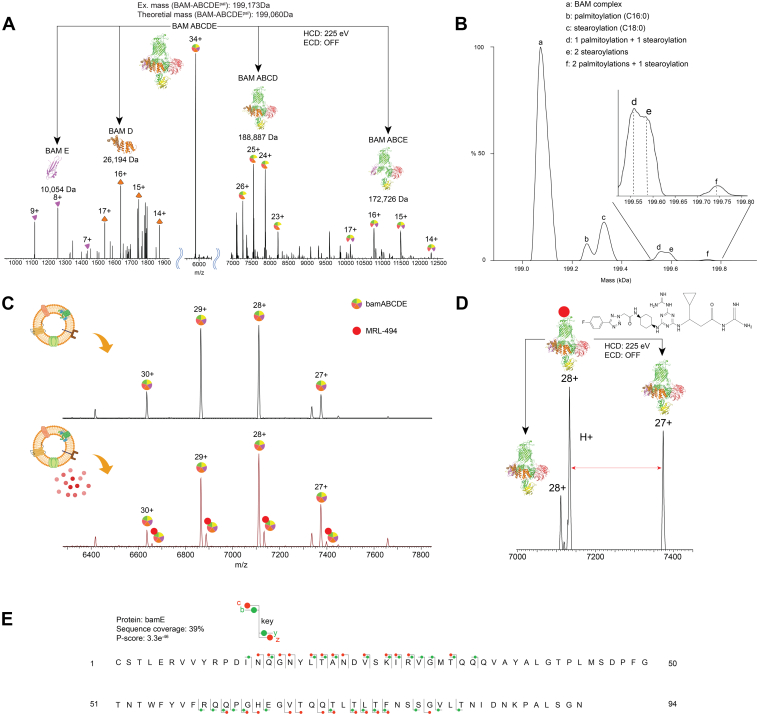
**nMS analysis of BAM complex and its ligand from *Escherichia coli* membrane vesicle.***A*, complex-up analysis of the 34+ charge state of the 199 kDa protein complex yields the subcomplex BAM–ABCD and ejected high-charged unfolded BAM E and subcomplex BAM ABCE through the ejection of monomeric BAM-D. *B*, deconvoluted high-resolution nMS spectra of the BAM complex show the presence of different protocorms arising through the S-acylation of BAM subunits. BAM B/C/D/E are all peripheral membrane proteins, where S-acylation has been reported as a mechanism for membrane anchoring. The mass additions observed match that of the most common fatty-acyl lipid chain in *E. coli* that is used for S-acylation. *C*, MS1 *E. coli* membrane vesicles treated with MRL494 (*bottom panel*). As a control, the *top panel* shows the same *m/z* region of the spectra of native vesicles, before the addition of MRL494. The spectra are zoomed in to focus on the BAM complex charge state regions. As seen, clear binding of the BAM complex and its inhibitor MRL494 (represented in *red*) is observed. *D*, to further confirm the binding, MRL494 bound a 28+ charge state of BAM complex, which was isolated and subjected to CID activation. This leads to the ejection of MRL494 from the complex with or without carrying a charge, generating both 27+ and 28+ apo BAM complexes. The difference calculated from the precursor to any of the apo-peaks again corresponds to the mass of MRL494. *E*, the MS2 data assignment for bamE for annotated terminal ion types is marked on the sequence, along with the *P* score. The HCD voltage and the status of the ECD cell are stated in each MS/MS spectrum. BAM, β-barrel–assembly machinery; CID, collision-induced dissociation; ECD, electron-capture dissociation; HCD, higher-energy collisional dissociation; nMS, native mass spectrometry.

Encouraged by our success of endogenous ligand binding to dimeric DLDH, we decided to pursue this route. For this experiment, we picked outer-membrane resident heteropentameric BAM complex that consists of IMP BAM-A and four other PMPs (BAM-B/C/D/E) (80, 81). This also coincides to evaluate our experimental approach on an IMP. In our fight against gram-negative bacteria, BAM complex, a chaperon of other outer membrane proteins, has emerged as a key target. BAM is essential to the stability of the outer membrane, and abrogating its function makes the bacteria susceptible. Critically, no currently marketed antibiotics target the BAM complex, making it a very promising target against antibiotic-resistant gram-negative bacteria. To perform the drug binding experiments on the BAM complex, our first target was to identify the BAM complex from the detected protein masses. Considering the theoretical mass of the heteropentameric BAM complex, we investigated the peaks that corresponded to the mass between 190 and 220 kDa. For ease of analysis, we first started with the complex-up analysis of putative peaks to check which one yields subcomplexes and ejected monomers that match the mass of the BAM subunits. Subsequently, the putative peaks were then confirmed through nTD analysis. Figure 5*A* shows the complex-up analysis of the peak at 5858 *m/z*, which upon CID activation yielded subcomplexes that exactly match with that of the BAM subunits. The data clearly show different asymmetric dissociation pathways of the overall heteropentamer. We could readily observe the ejected monomeric BAM-D (mass = 26193 Da), with the remainder of the intact tetramer of BAM-ABCE (mass = 172,525 Da). Further, we could also observe ejection of the monomeric subunit E (mass = 10054 Da) leading to the generation of the intact tetrameric BAM-ABCD with charge states ranging from 23 to 26 (mass = 188,887 Da). In both these cases, the charges partitioned between the unfolded monomer and folded tetramer add up to the parent pentamer, further strengthening the assignment.

Interestingly, we could also see specific proteoforms that are necessary for the association of the BAM complex with the biological membranes. While BAM-A is an IMP, other subunits of BAM are PMPs that are known to be lipidated (80). Figure 5*B* shows deconvoluted masses of the BAM complex proteoforms that differ by specific lipidation masses. The mass addition corresponds to the addition of palmitate and oleic fatty acyl chains, which are two of the most abundant fatty-acyl chains in the *E. coli* membrane. While the complex-up data were very confirmatory to establish the species as the BAM complex before performing the drug binding experiments, we also confirmed this through top–down analysis of the subcomplexes (supplemental Fig. S7).

While the initial attempt to block the BAM complex was through designing antibodies, recent discoveries of natural and synthetic molecules have ignited possibilities of finding small-molecule drugs that can target BAM (82, 83). For the current study, we picked MRL494 as our target candidate (83). MRL494 is a synthetic small molecule that is shown to have antibacterial activity. Mutant screening experiments deduced that mutation in BAM-A (E470K) can confer resistance to MRL494 and subsequent thermal screening indicating binding of MRL494 proximal to BAM complex. Nevertheless, subsequent thermal screening of MRL494 resistance mutant BAM (BAM-A/E470K) showed no change in the thermal shift. Further, MRL494 was also observed to act on the inner membrane with possible antibiotic effects. Together, this shrouds an ambiguity over BAM as the possible site of direct binding of MRL494, and to date, no direct evidence of MRL494 binding to BAM has been observed. Exploiting our ability to detect the BAM complex from the native membrane, we attempted to see if we could directly observe the binding of MRL494 to BAM-A.

*E. coli* vesicles were treated with 100 μM MRL494 and incubated briefly on ice. nMS analysis of these vesicles showed the emergence of new satellite peaks for all BAM-complex charge states, which corresponds to BAM–MRL494 complex mass. As a control, Figure 5*C* (*top panel*) shows the same *m/z* section of the nMS spectra of just the native vesicle, without incubating with MRL494. None of the putative MRL494-bound peaks was observed. To further confirm the peak is indeed MRL-494-bound species, the 28+ charge state of the putative BAM–MRL494 species was isolated and subjected to complex-up analysis. MRL-494 ejection with and without a charge could be clearly observed, resulting in both 27+ and 28+ charge states of unbound apo-BAM complex. The results provide direct evidence for MRL-494 BAM complex interaction taking place in the native physicochemical environment in which the BAM complex resides.

## Discussion

MS-based analysis of IMPs initiated with LC-based separation followed by intact mass measurements of individual IMPs (10, 11, 14, 46). This enabled both high-resolution mass determination of intact protein subunits in multiprotein complexes and identification of these individual units through top–down MS. But since LC-based separation of proteins typically leads to their denaturation, the missing technical milestone determined the native oligomeric masses of the formed MP complexes. In subsequent years, the introduction of nMS enabled the detection of MPs, in their complex states with other proteins, lipids, and ligands in the oligomeric organization states. These developments further pushed technological demand to perform a similar analysis but directly from the physiological membranes. We previously showed that with the help of superchargers, MP–lipid complexes can be directly detected from tunable *in vitro* liposomes. Here, we further extend the platform that now enables us to perform nTD analysis of MP complexes directly from the lipid membrane. We achieve this by first ablating the lipid bilayer–embedded MPs in the prequadrupole front-end section of MS, isolating the desired *m/z* at the quadrupole and performing top–down fragmentation by combining nonergodic ECD and ergodic CID fragmentation. First using model IMPs, we show how this could be used to ID MPs, directly from the lipid membrane, through nTD analysis of their physiological oligomeric states. We subsequently employed this nTD and complex-up analysis on native membrane vesicles to perform nTD proteoform-ID and complex-up-based stoichiometry determination of an MP and a peripheral protein complex directly from their native membranes. Further, we show how this approach could be used to study endogenous flavin adenine dinucleotide binding to DLDH and exogenous ligand binding/screening (MRL494 for BAM) directly on the native membrane.

It is noteworthy that despite utilizing both ECD and HCD fragmentation techniques, a substantial proportion of the detected ions were of the conventional b and y type. We think this is because, in most EChcD experiments, high levels of HCD energy were necessary to achieve effective fragmentation spectra. We attribute this to the requirement of breaking extensive hydrogen-bonding networks that continue to hold dissociated fragments together post-ECD. Thus, collisional activation is essential to disrupt these noncovalent interactions and release detectable fragment ions. This necessity mirrors the concept of supplemental activation, which is found to significantly enhance ECD fragment ion abundance in peptide fragmentation spectra (67). However, here we are fragmenting large oligomeric complexes, which inherently possess more extensive noncovalent interactions maintaining their native structural integrity. Consequently, even higher CID energies are required to overcome these interactions. Indeed, except for the relatively small protein VAMP2 (12 kDa), all our EChcD experiments utilized HCD energies exceeding 150 eV. We propose that these elevated CID conditions likely promote extensive b and y ion fragmentation. Further, they may also trigger secondary fragmentation yielding c- and z-type ions, thus creating internal ions. Currently, however, top–down database search software primarily evaluates terminal ions. This leaves the generated internal ions unannotated. Addressing this limitation will require future developments of advanced data analysis frameworks capable of incorporating both terminal and internal ion types while maintaining robust statistical validation of matched ions.

One limitation of the current work is while the top–down data acquisition was done at high resolution (between 100,000 and 200,000), most MS1 acquisitions were done at relatively low resolution, especially for the native membrane vesicle. This is because high-resolution data acquisition increases the transient time in orbitrap-based data acquisition (84). This in turn means increasing resolution comes at the expense of losing sensitivity in detecting high molecular weight ions, as they tend to die off sooner compared with the lower molecular weight counterparts. Hence, while performing nMS on large complexes in orbitrap-based instruments, MS1 is typically acquired at low resolution to obtain the best possible signal (85). Following this, while detecting ablated protein masses from the native vesicles, we picked lower resolutions that provide the best opportunity to detect a wide range of masses, going up to almost half a megadalton. This in turn precludes high-resolution mass determination of IMP complexes. Nevertheless, recent developments in orbitrap-based single-ion mass spectrometric approaches provide an excellent avenue to mitigate this limitation. Application of this approach on nMS of soluble protein complexes has demonstrated its utility in determining high-resolution masses of protein complexes (25, 86). We expect future work to demonstrate similar capabilities while studying MPs directly from native membrane vesicles.

A point to note is that despite our best attempts we have not been able to identify the protein complex around 466 KDa. We think this is owing to its very large size, which makes it recalcitrant to direct top–down ID. A possible avenue here is to perform mutistage tandem MS experiments where the first round of complex-up analysis can yield monomeric subunits, which can then be quadrupole isolated and subjected to top–down fragmentations. The current instrument framework used does not provide such capabilities, as the front-end in-source trapping-CID is used up to ablate the proteins from the bilayer. Nevertheless, other instrumentation platforms possibly capable of such avenues can certainly provide an exciting prospect to alleviate the need.

Another obvious limitation of this work arises from ion suppression in electrospray ionization (87). Since we do not have any chromatographic separation, the entire proteome associated with the total membrane flies into the MS and competes for the limited supply of protons available in the droplets. This skews the detection toward more abundant or easily ionizable proteins. Here, IMPs are at a disadvantage over peripheral proteins (PMPs) that are associated with the membrane either through their direct membrane binding or through binding with other MPs. This is because IMPs have a significant portion of their surface area embedded within the membrane and have fewer polar basic residues compared with peripheral MPs. Consequently, while applying this approach on native membranes containing the entire membrane-associated proteome, PMPs are expected to be more readily detectable over their integral counterparts.

Nevertheless, many IMPs have either large cytosolic domains or their prevalent physiological state in the membrane is a multiprotein complex state with other PMPs. Either of these cases should also provide these classes of IMPs a competitive chance for detection by capturing protons. This is evident in the *E. coli* membrane vesicle spectra as we see the BAM complex, which contains an IMP BAM-A in complex with four PMPs, but not more abundant outer membrane protein-A, which is an IMP that contains very little surface area exposed outside the membrane. We of course did not have this problem in liposomes, where only the target IMPs–PMPs are reconstituted but not the entire membrane proteome. This brings us to the obvious next step in the method development, that is, how do we fine-tune the method to evolve into a selective approach that can be applied to study any target IMP–PMP from the native membrane but not just the most prevalent/ionizable fraction of the proteome.

Taking a cue from our liposome-based work, a plausible solution is to not subject the total membrane containing the entire proteome to MS but only the membrane nanodomain that contains the target IMP–PMP. This would require upstream biochemical intervention to excise out and enrich precisely the nanodomain of a whole cell membrane that contains a target protein of interest, along with its natively bound lipids and proteins in a complex state. Using a class of membrane-active polymer, we have recently developed a chemical biology approach that enables targeted enrichment of the nanoscale native membrane domain of target IMP–PMP, directly from a whole cell membrane. Using these membrane-active polymers, we have further developed a high-throughput platform to perform the enrichment for more than 2000 individual MPs (88). This enables us to specifically enrich only the membrane nanodomains that contain a target IMP–PMP and its complexes and subject it to downstream MS, instead of the total cell membrane.

At the next stage, we envision a unison of such chemical biology approach with the current work to yield a targeted membrane biology platform to study any target MP and its complexes directly from its native cellular membranes, where the work described here will provide the technological roadmap to detect and ID endogenous protein complexes through nTD analysis, directly from the membrane.

## Data availability

All the .raw data associated with this article has been deposited in figshare and can be found here—https://figshare.com/articles/dataset/_b_Native_Top-down_analysis_of_membrane_protein_complexes_directly_from_b_b_i_in_vitro_i_b_b_and_native_membranes_b_/28216862.

## Supplemental data

This article contains supplemental data (15, 35, 47, 48, 49, 50, 54, 89, 90, 91)(http://patternlabforproteomics.org/yada3/#_Toc108263859).

## Conflict of interest

The authors declare no competing interests.
