# Supplementary material for: Native Top–Down Analysis of Membrane Protein Complexes Directly From In Vitro and Native Membranes
Source: Mol Cell Proteomics. 2025 May 14;24(7):100993. doi: 10.1016/j.mcpro.2025.100993 (PMC12305242; doi:10.1016/j.mcpro.2025.100993)
Supplement: Supplemental data [file mmc1.docx]

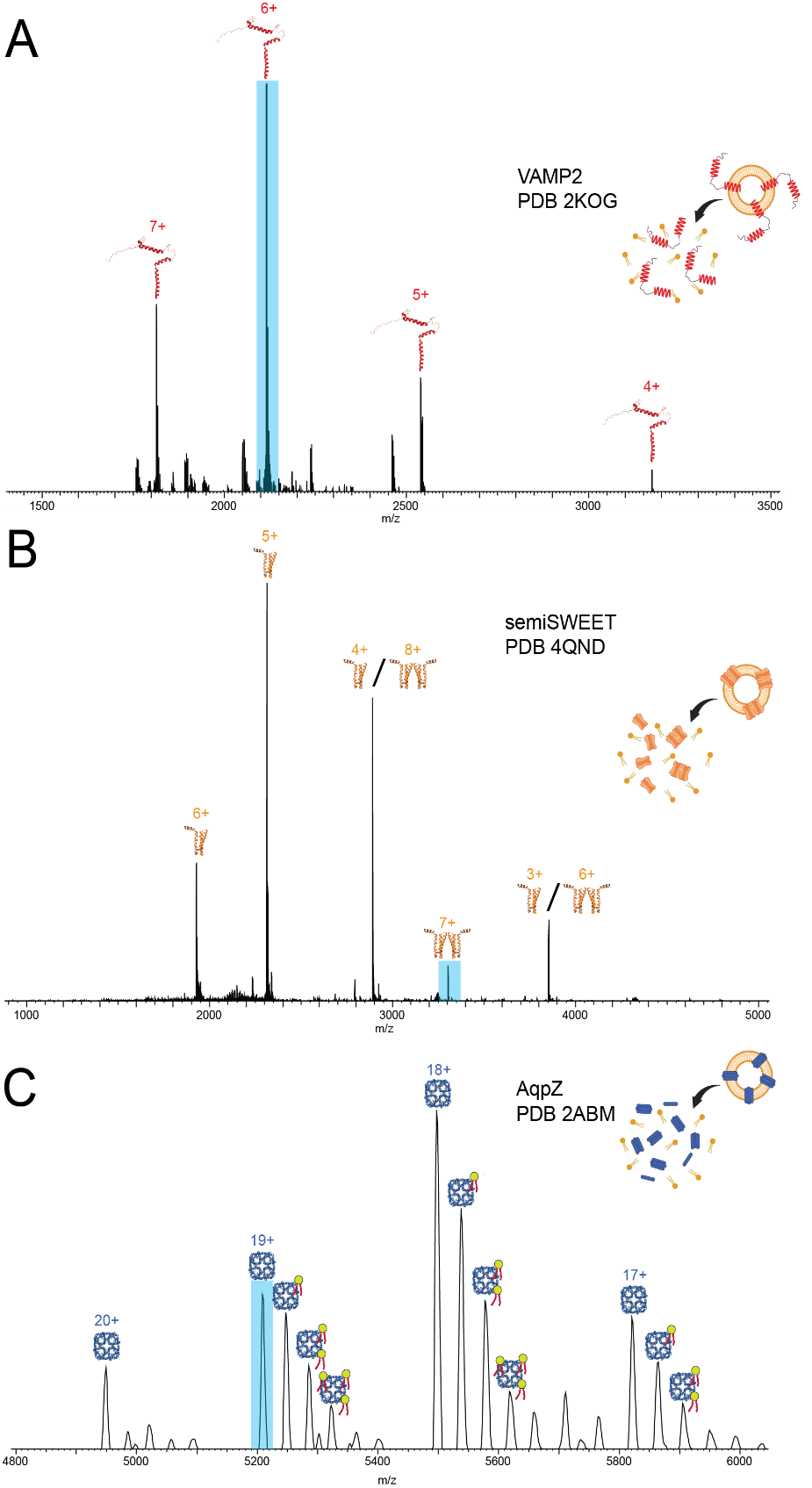


**SI Figure 1:** **Membrane proteins in their physiological oligomeric state detected from *in vitro* liposome.** Following the previously established protocol, VAMP2, semiSWEET, and AqpZ were incorporated into *in vitro* liposomes and subjected to nMS. The resulting MS1 spectra of VAMP2 (A), semiSWEET (B), and AqpZ (C), depicting their native oligomeric states, are shown here. The 6+ charge state of the VAMP2 monomer, the 7+ charge state of the semiSWEET dimer, and the 19+ charge state of AqpZ tetramer are highlighted (as in cyan) as they were subjected to nTD analysis via EChcD.


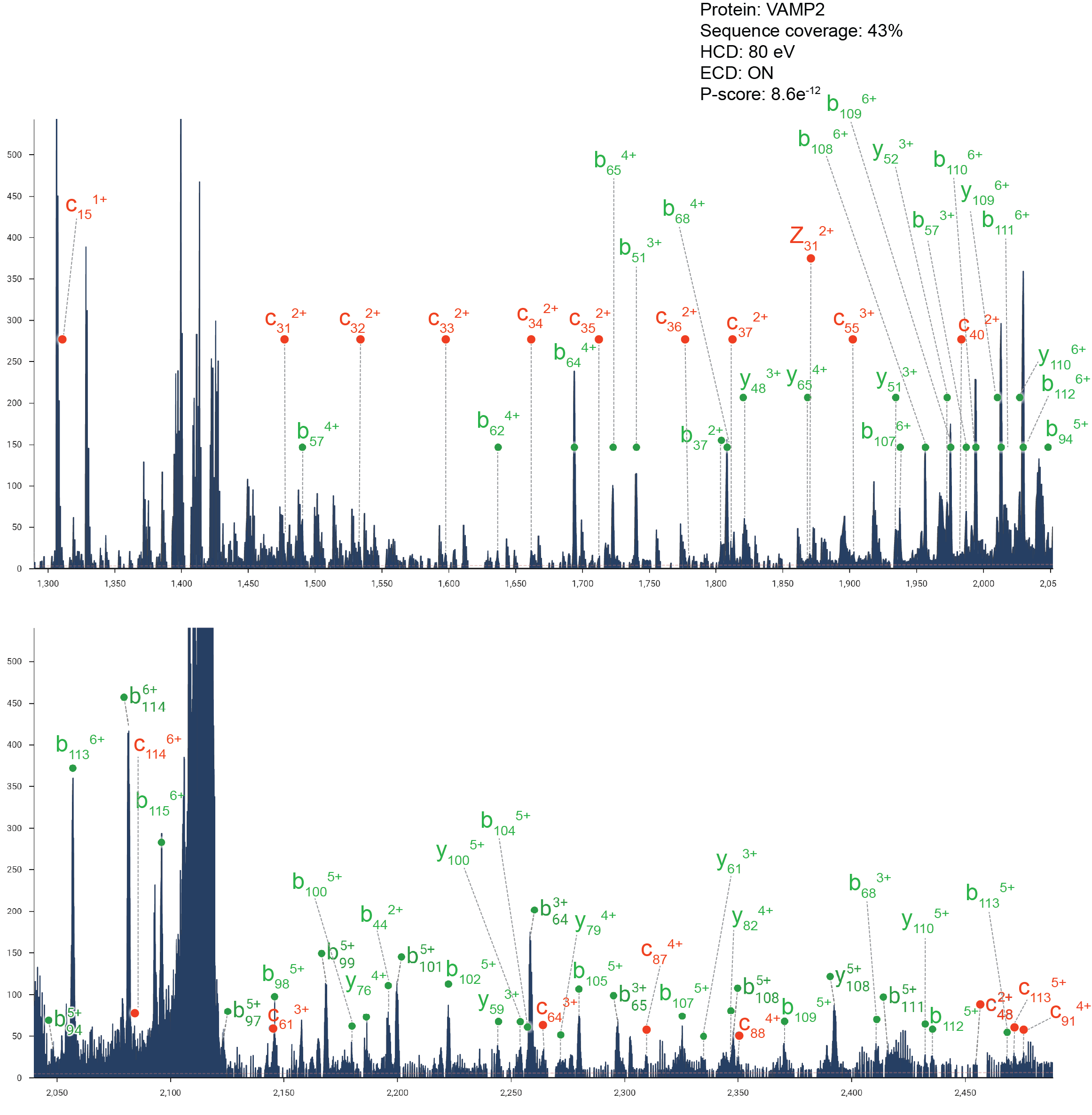


**SI Figure 2:** Expanded annotation of nTD spectra of VAMP2 directly from proteoliposome. The spectra were obtained by ablating VAMP2 from the liposome and subjecting the 6+ charge state to EChcD-based nTD fragmentation. This figure, along with Figure 2B, shows the annotations of the terminal ions observed. Overall, 43% sequence coverage was obtained. The HCD voltage and the status of the ECD cell is stated in the MS/MS spectra


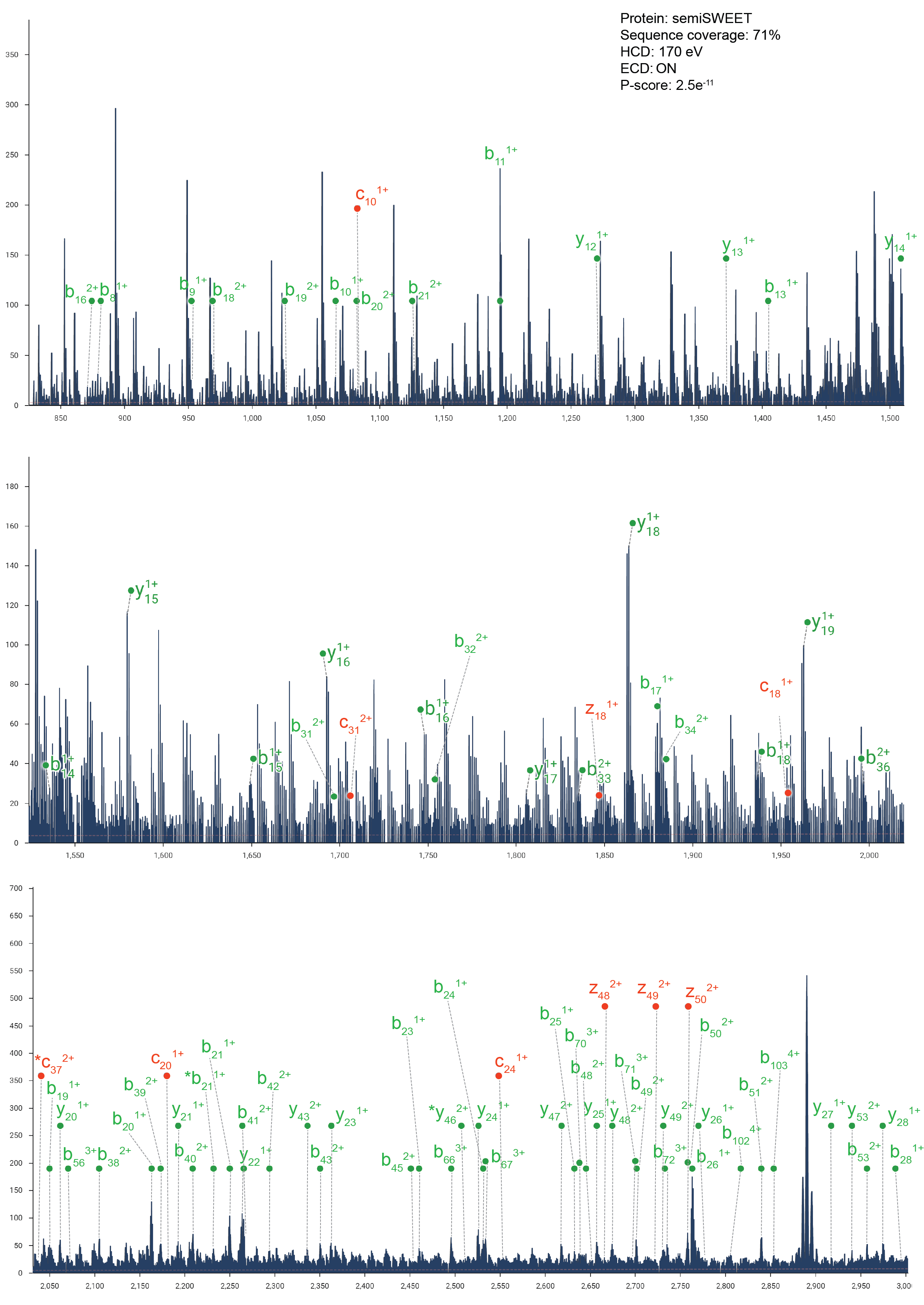


**SI Figure 3:** Expanded annotation of nTD spectra of dimeric semiSWEET directly from the proteoliposome. The spectra were obtained by ablating semiSWEET from the liposome and subjecting the 7+ charge state of the dimer to EChcD-based nTD fragmentation. This figure, along with Figure 2C, shows the annotations of the terminal ions observed. Overall, 71% sequence coverage was obtained. The HCD voltage and the status of the ECD cell is stated in the MS/MS spectra.

**SI Figure 4:** Expanded annotation of nTD spectra of AqpZ directly from the proteoliposome. The spectra were obtained by ablating AqpZ from the liposome and subjecting the 19+ charge state of the tetramer to EChcD-based nTD fragmentation. This figure, along with Figure 2D, shows the annotations of some of the terminal ions observed. Overall, 30% sequence coverage was obtained. Three different neutral losses, NH_3_ (denoted with #), CONH_2_ (denoted with ##), and H_2_O (denoted as *), were included. The HCD voltage and the status of the ECD cell is stated in each MS/MS spectra


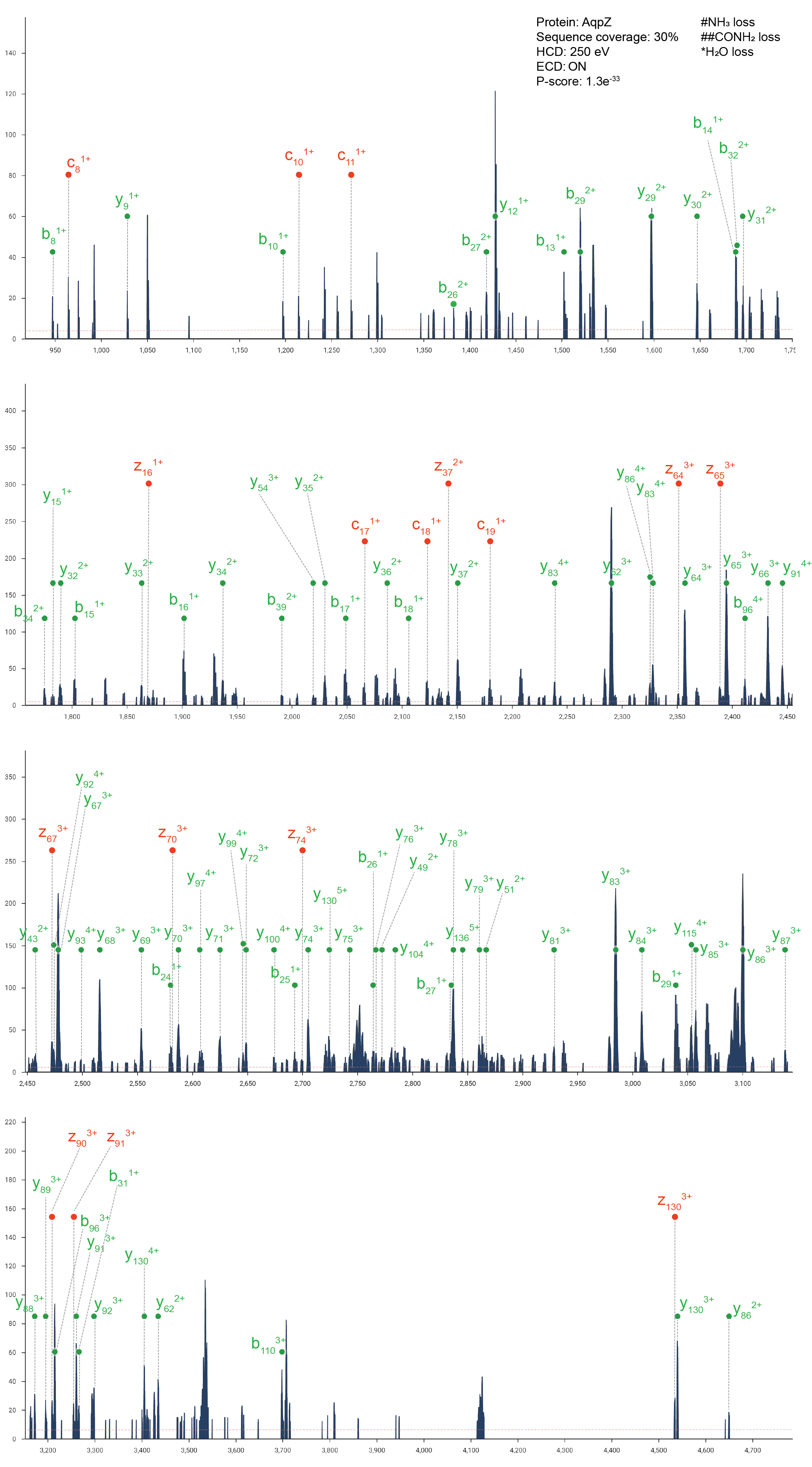


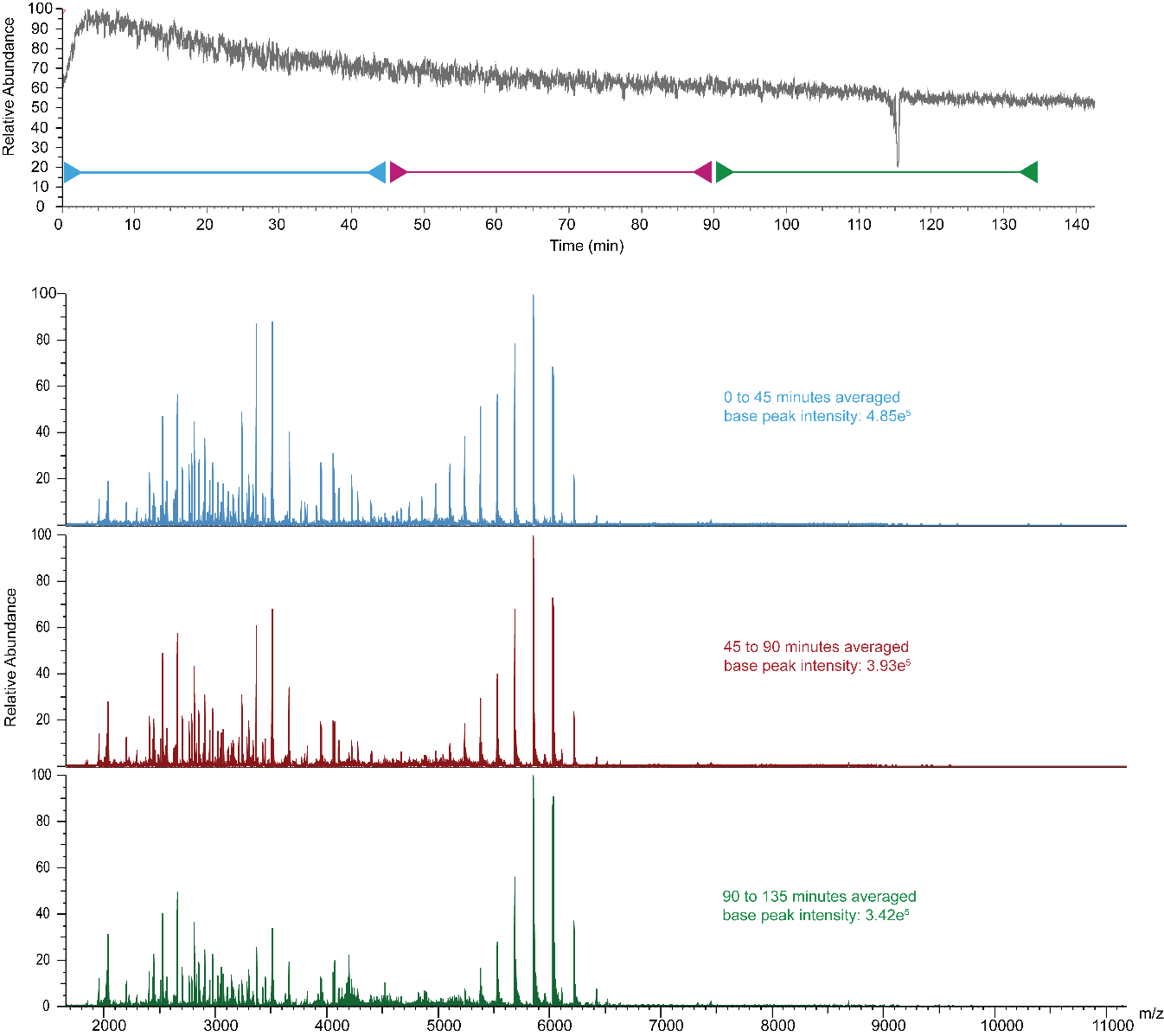


**SI Fig. 5: Membrane vesicle nESI signal stability.** The nMS-ready vesicles prepared from the *E. coli* membrane were treated with a supercharging agent (5% v/v glycerol) and then analyzed with the same conditions as shown in Fig. 1C. Nano ESI emitter with an opening diameter of approximately 4.5um was used to spray 5ul of the sample (approximately 300ug/ml total protein concentration). The spray lasted longer than 140 minutes, with the (**A**) TIC intensity decreasing by approximately 35% during the whole period. The averaged signal from 0 to 45 minutes (in cyan) (**B**), 45 to 90 minutes (in dark red) (**C**), and 90 to 135 minutes (in dark green) (**D**) are shown here along with their respective base peak intensity. The spectra pattern remains relatively constant throughout the nESI duration, providing sufficient signal stability necessary for nTD-MS analysis.


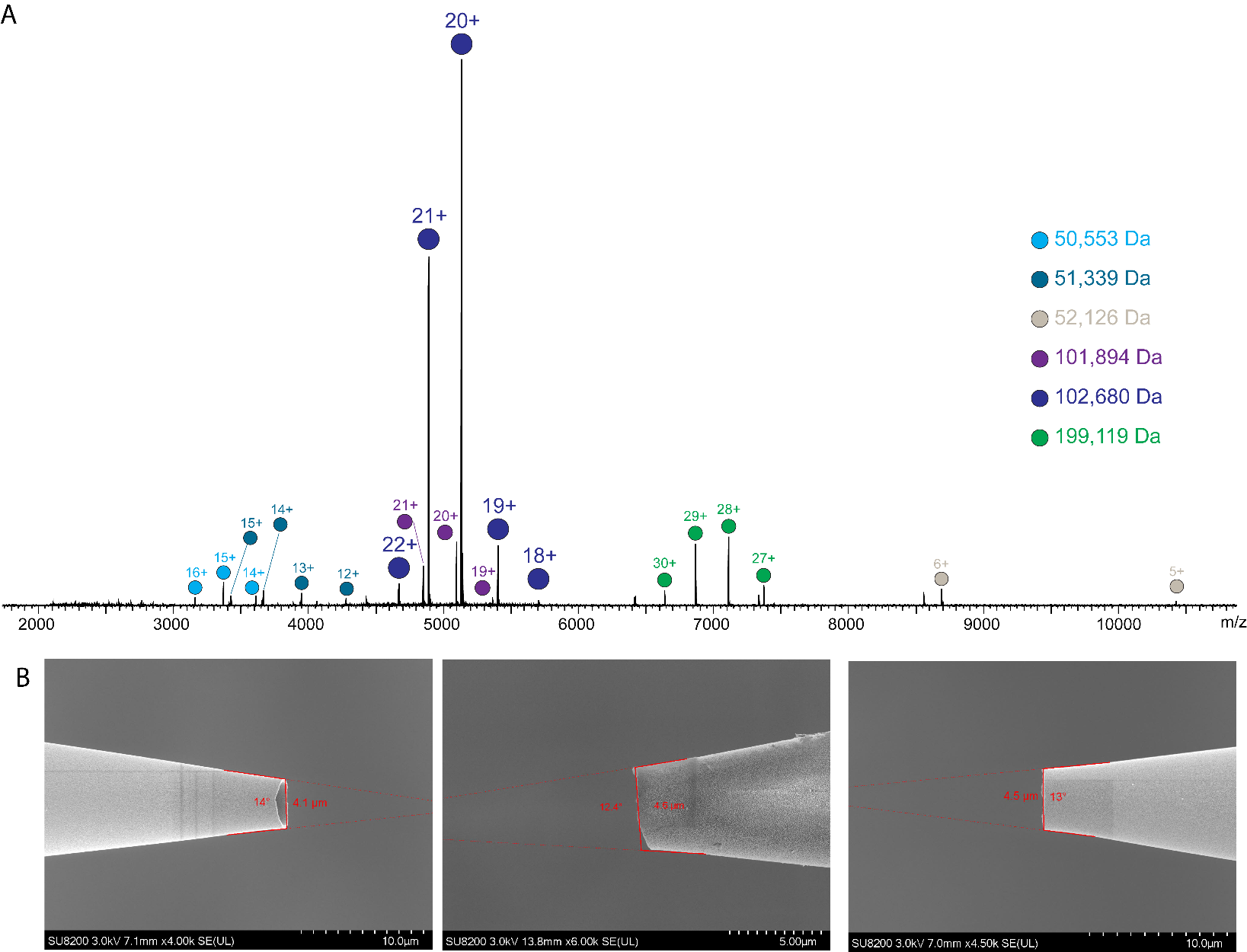


**SI Fig. 6:** **nMS of E coli membrane vesicles without supercharging agents.** **(A)** nMS of *E. Coli* membrane vesicles prepared from *E. coli* membrane were recorded under similar MS conditions as shown in Fig. 1C but without a supercharging agent. Signal intensities of ejected protein are considerably lower, with fewer proteins detected, when compared to supercharging conditions. (**B**) The SEM pictures of three independent pulled capillaries, with their opening diameters ranging from 4 to 4.8 μm and the taper angle ranging from 12-15 degrees.


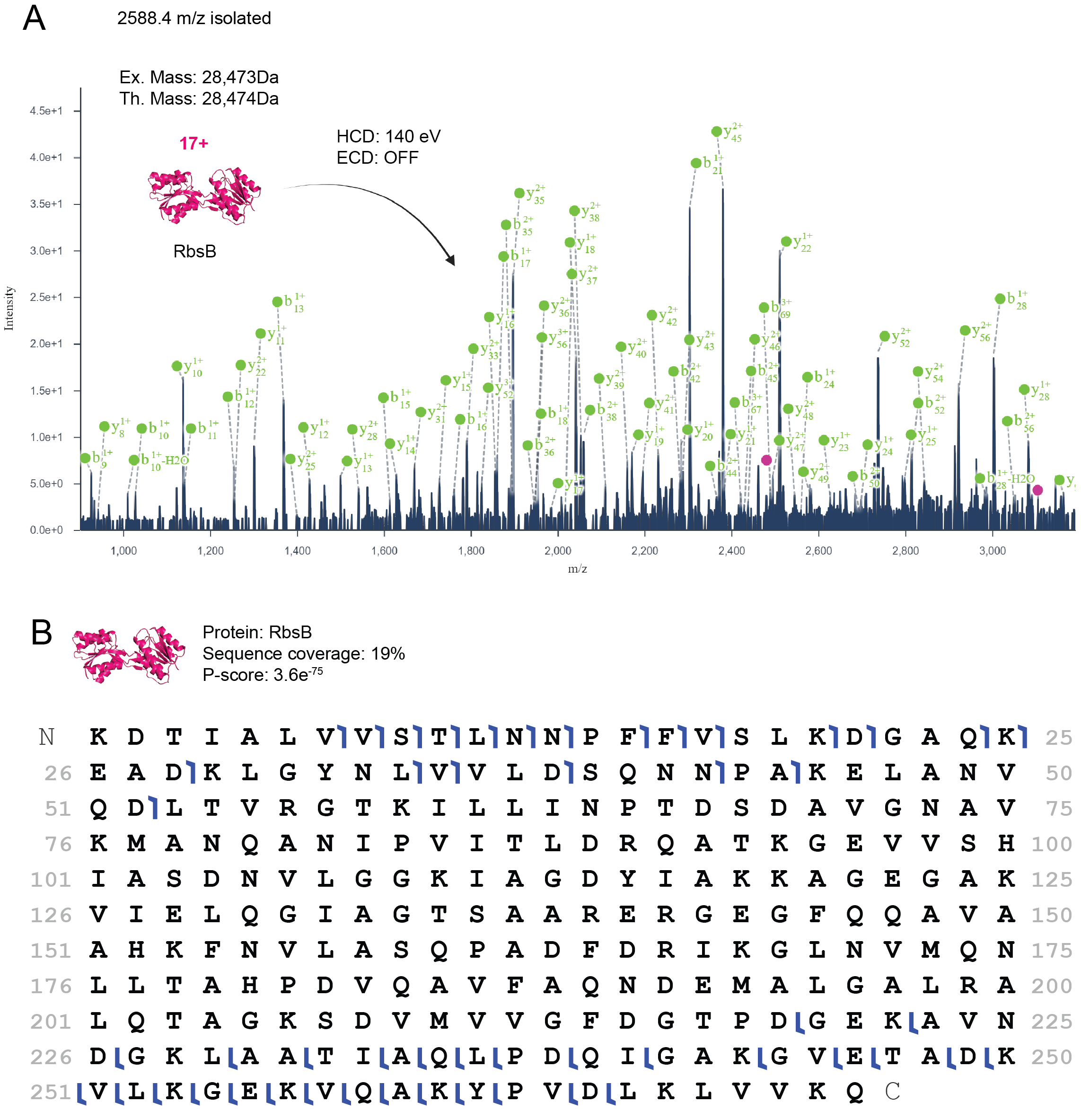


**SI Fig. 7: Identification of RbsB protein from membrane vesicle using nTD.** The nMS-ready vesicles prepared from *E. coli* membrane were treated with a supercharging agent (5% v/v glycerol) and then analyzed under the same conditions as shown in Fig. 1C. (A) The experimental mass of the protein is 28,473 Da, and the theoretical mass of the RbsB protein is 28,474 Da. Expanded annotation of nTD spectra of RbsB directly from membrane vesicles is shown. The spectra were obtained by ablating RbsB from membrane vesicles and subjecting the 17+ charge state to HCD-based nTD fragmentation. The b/y fragments are highlighted in green and the c/z fragments in red. (B) The MS2 data was searched against the database that includes the total *E. coli* proteome. The P-score is displayed here along with the sequence coverage. The HCD voltage and the status of the ECD cell is stated in each MS/MS spectra

**SI Fig. 8: Identification of Superoxide dismutase [Fe] protein from membrane vesicle using nTD.** The nMS-ready vesicles prepared from *E. coli* membrane were treated with a supercharging agent (5% v/v glycerol) and then analyzed with the same conditions as shown in Fig. 1C. (A) The experimental mass of the protein is 42,266 Da and the theoretical mass of Superoxide dismutase [Fe] protein is 42,268 Da. Expanded annotation of nTD spectra of Superoxide dismutase [Fe] directly from membrane vesicles is shown. The spectra were obtained by ablating Superoxide dismutase [Fe] from membrane vesicles and subjecting the 12+ charge state to HCD-based nTD fragmentation. The b/y fragments are highlighted in green and c/z fragments are in red. (B) The MS2 data was searched against the database that includes the total *E. coli* proteome. The P-score is displayed here along with the sequence coverage. The HCD voltage and the status of the ECD cell is stated in each MS/MS spectra.


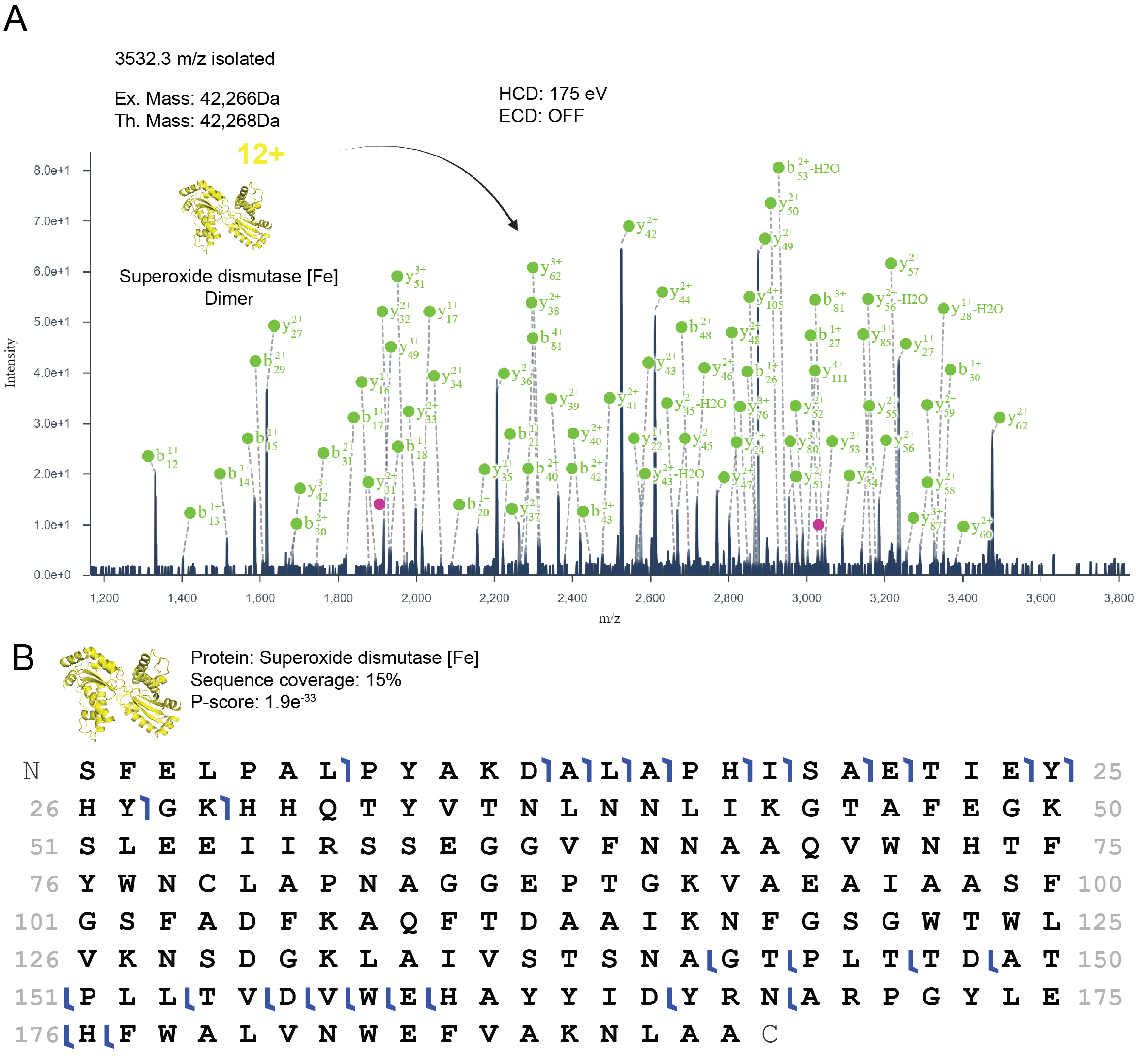


**SI Fig. 9: Identification of Tryptophanase protein from membrane vesicle using nTD.** The nMS-ready vesicles prepared from *E. coli* membrane were treated with a supercharging agent (5% v/v glycerol) and then analyzed with the same conditions as shown in Fig. 1C. (A) The experimental mass of the protein is 105,540 Da, and the theoretical mass of Tryptophanase protein is 105,542 Da. Expanded annotation of nTD spectra of Tryptophanase directly from membrane vesicles is shown. The spectra were obtained by ablating Tryptophanase from membrane vesicles and subjecting the 24+ charge state HCD-based nTD fragmentation. The b/y fragments are highlighted in green, and c/z fragments are in red. (B) The MS2 data was searched against the database that includes the total *E. coli* proteome. The P-score is displayed here along with the sequence coverage. The HCD voltage and the status of the ECD cell is stated in each MS/MS spectra


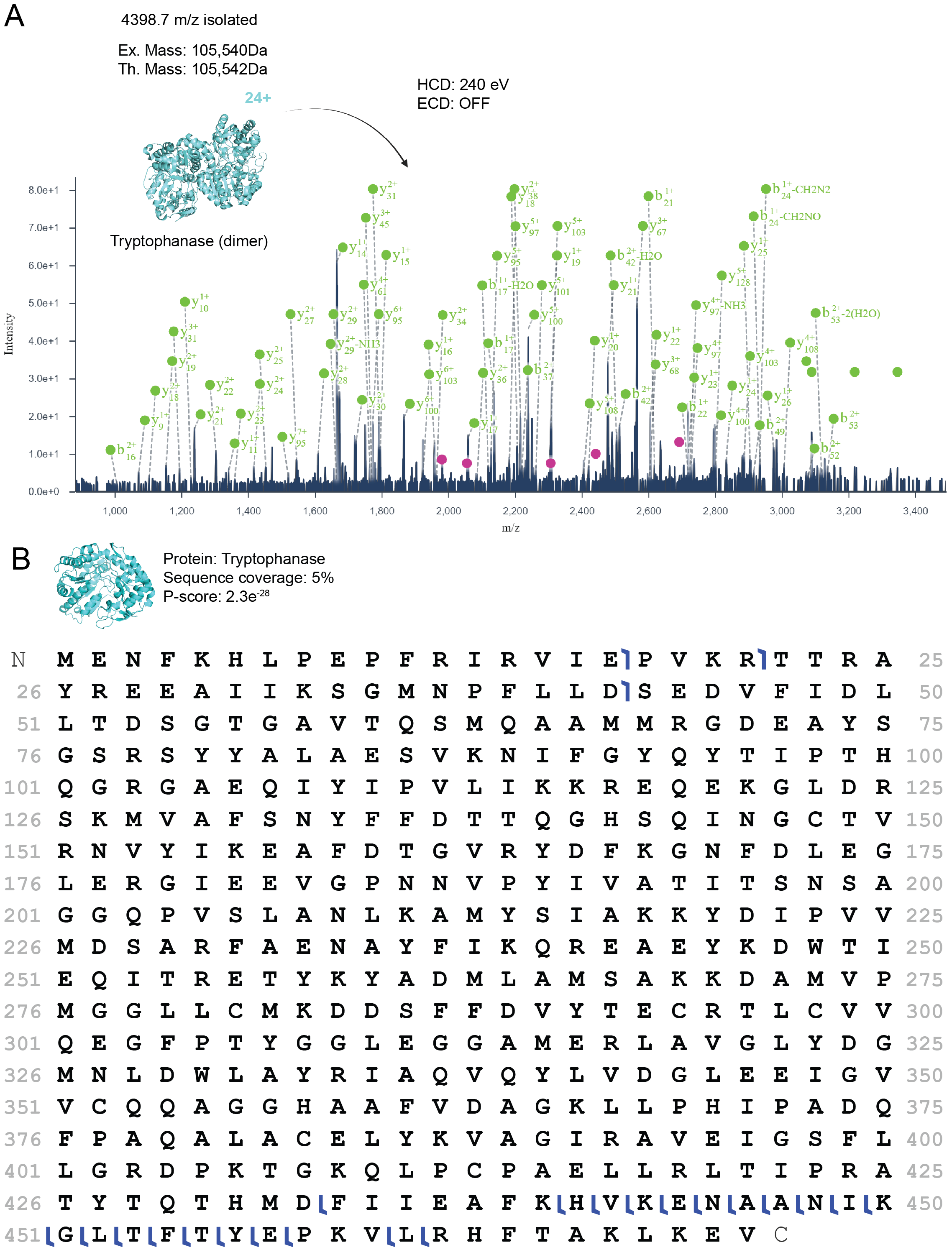


**SI Fig. 10: Identification of DLDH (monomer) protein from membrane vesicle using nTD.** The nMS-ready vesicles prepared from *E. coli* membrane were treated with a supercharging agent (5% v/v glycerol) and then analyzed with the same conditions as shown in Fig. 1C. (A) The experimental mass of the protein is 50,556 Da, and the theoretical mass of DLDH monomer protein is 50,557 Da. Expanded annotation of nTD spectra of DLDH monomer directly from membrane vesicles is shown. The spectra were obtained by subjecting the 17+ charge state of the DLDH monomer to HCD-based nTD fragmentation. The b/y fragments are highlighted in green, and c/z fragments are in red. (B) The MS2 data was searched against the database that includes the total *E. coli* proteome. The P-score is displayed here along with the sequence coverage. The HCD voltage and the status of the ECD cell is stated in each MS/MS spectra


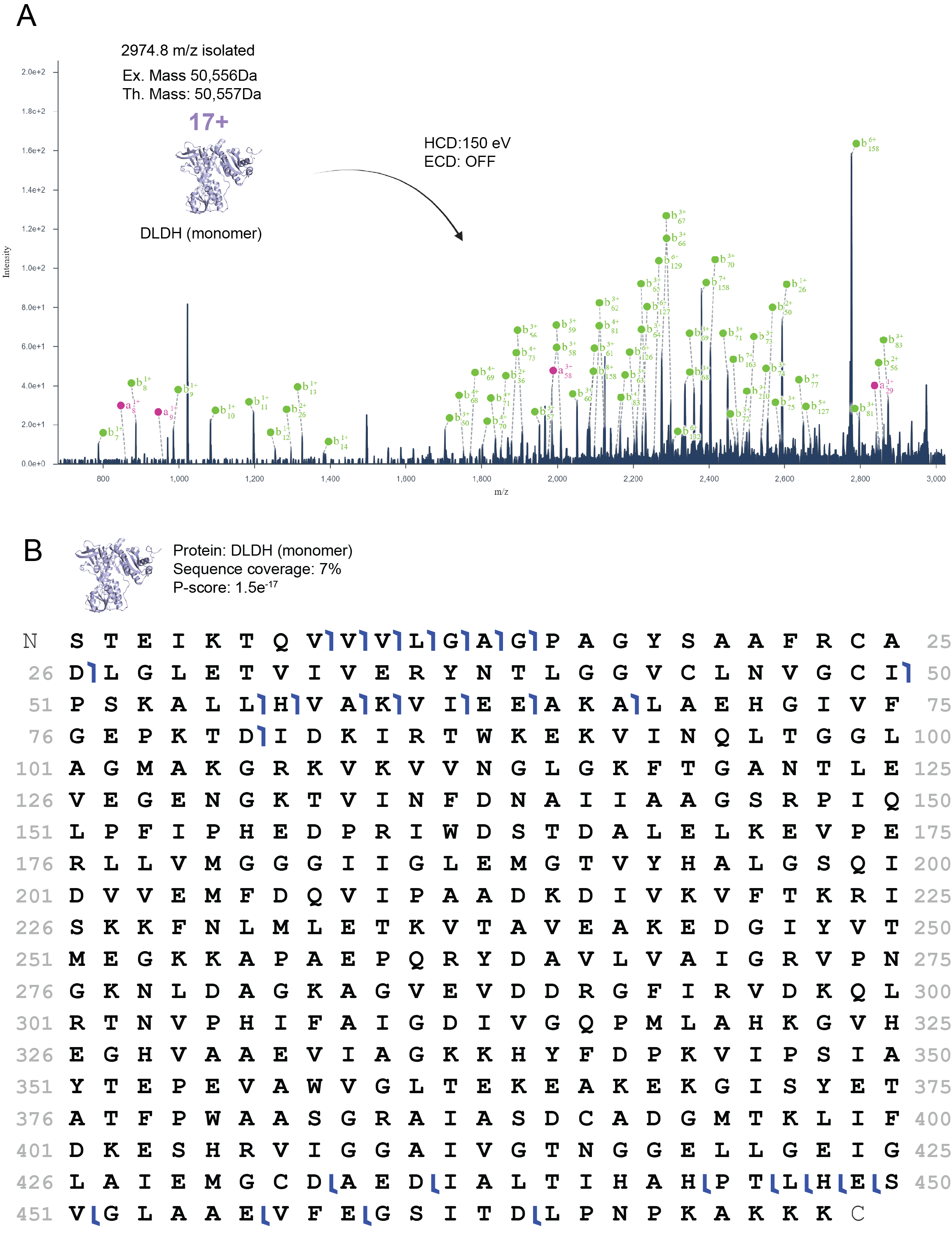


**SI Fig. 11:** **nTD-MS analysis of BAM complex.** (A) *E. coli.* membrane vesicle solution treated with 2% NH_4_OH leads to partial dissociation of the BAM complex leading to the disappearance of the 199kDa BAM complex peak and generation of BAM AE sub-complex (99.6kDa, BAM A and palmitoylated BAM E). The top panel shows the spectra of the vesicle before NH_4_OH addition, and the bottom panel after. (B) The 19+ charge state of the 99KDa BAM-AE complex was subjected to nTD analysis via EChcD, and a section of the spectra with annotated terminal ions are shown here. This resulted in 41% and 3% sequence coverage for BAM E and BAM A, respectively. The fragments from BAM E are highlighted in green and fragments from BAM A in blue. The unannotated peptide peaks are from PDH E1 component (Uniprot ID: P0AFG8 · ODP1_ECOLI), which are co-isolated during the quadrupole isolation due to the mass similarity (theoretical mass of BAM AE: 99599 Da, theoretical mass of PDH E1 monomer: 99537 Da)


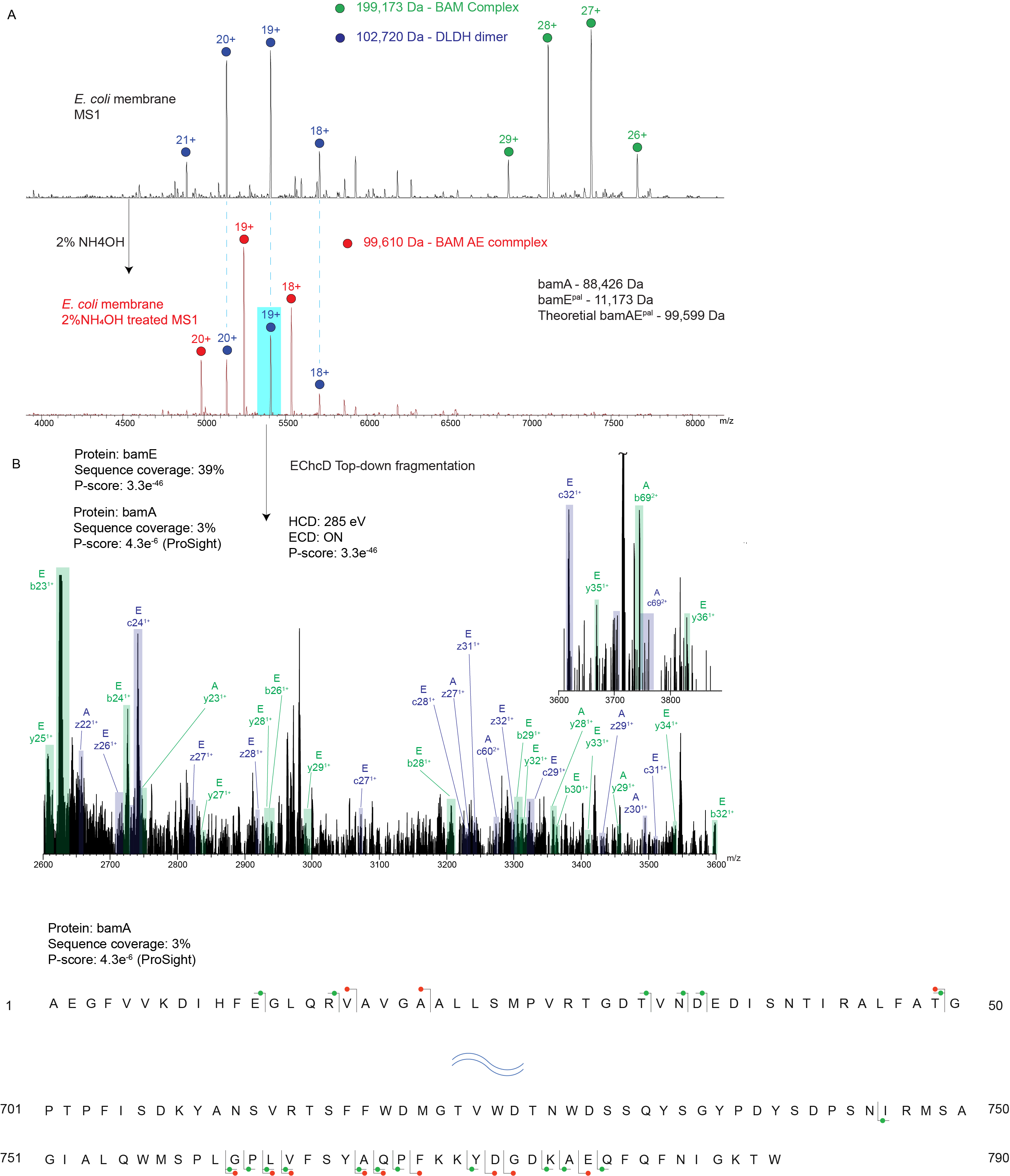


**SUPPLEMENTARY METHODS**

**A. Details on expression and purification of recombinant membrane proteins:** Recombinant v-SNARE (VAMP2) was expressed and purified as previously described[^1–4^](https://sciwheel.com/work/citation?ids=14760934,53203,53224,235735&pre=&pre=&pre=&pre=&suf=&suf=&suf=&suf=&sa=0,0,0,0&dbf=0&dbf=0&dbf=0&dbf=0). Briefly, the protein was expressed in the *E. coli* BL21 strain using 0.5mM isopropyl β-D-1-thiogalactopyranoside (IPTG) for 4 h at 37°C. Cells were centrifuged and lysed using a cell disruptor (Avestin) in HEPES buffer (25mM HEPES, 400mM KCl, 4% Triton X-100, 10% glycerol, pH 7.4) containing 1 mM dithiothreitol (DTT). Cell debris was pelleted by centrifugation (142,100g, 30 minutes), and the supernatant was subsequently incubated with Ni-NTA resin (Thermo Fisher Scientific) overnight at 4 °C. After being washed with HEPES buffer supplemented with 15 mM imidazole, 1% OG, 1mM DTT, and then with HEPES buffer supplemented with 25mM imidazole, 1% OG, 1mM DTT, pH 7.4, the resin was incubated with small ubiquitin-like modifier (SUMO) protease in a HEPES buffer enriched with 1% OG and 1mM DTT overnight at 4 °C. The protein was eluted with the gravity flow column on the next day.

AqpZ and semiSWEET were expressed and purified as described previously[^1,5,6^](https://sciwheel.com/work/citation?ids=14760934,464418,2992956&pre=&pre=&pre=&suf=&suf=&suf=&sa=0,0,0&dbf=0&dbf=0&dbf=0). Briefly, 20 ml of Rosetta (DE3)pLysS cells with plasmids encoding semiSWEET or AqpZ were grown overnight. The seed cultures were transferred to fresh culture and the bacteria were grown until optical density (OD) 600 reached 0.8. Then, AqpZ was expressed for 4h at 37 °C and semiSWEET for 15h at 22 °C with 0.2 mM IPTG. Cells were collected by centrifugation (4,000g, 10 min, 4 °C) and then resuspended in 100 ml of lysis buffer (20 mM Tris, 300 mM NaCl, pH 7.4) supplemented with protease inhibitor cocktail tablets (Pierce protease inhibitor mini tablets). Cells were lysed using a cell disruptor (Avestin), and cell debris was removed by centrifugation (20,000g, 20 min, 4 °C). From the supernatant, the membranes were pelleted by ultracentrifugation (100,000g, 2 h 15 min, 4 °C). Then, the membranes were homogenized in 30ml ice-cold membrane resuspension buffer (20 mM Tris, 100 mM NaCl, 20% glycerol, pH 7.4) using a Potter-Elvehjem Teflon pestle. Then, the homogenized membranes were treated with powder DDM to the final concentration of 2% (wt/vol) and were left to tumble at 4 °C for 2h. Insoluble material was cleared by centrifugation (20,000g, 40 min, 4 °C), and then the supernatant was filtered through 0.22-µm filters. His-tag affinity chromatography was performed in 20mM Tris, 150mM NaCl, 10% glycerol, 0.02% DDM, and pH 7.4 for the first round of purification. Then, bound semiSWEET and AqpZ were cleaved with PreScission Protease and TEV protease, respectively. For the second round of purification, reverse Ni chromatography and then size-exclusion chromatography (SEC) were performed. The detergent was exchanged to OG during SEC for both proteins.

**B preparing nanoelectrospray needles:** The nanoelectrospray capillaries are formed by pulling borosilicate glass capillaries (outer diameter: 1.5mm, inner diameter: 1.1 mm, length: 7.5cm; Sutter Instruments) using a Flaming/Brown micropipette puller (Model P-1000, Sutter Instruments) equipped with the 3.0mm by 3.0mm square box filament (Sutter Instruments). The puller’s parameters were fine-tuned to achieve an approximate opening diameter of 4.5um, and the taper angle around 15 degrees for the final emitter capillaries (SI Fig. 6). The exact measurements were performed by scanning electron microscopy with Hitachi SU8230 CFE SEM with BRUKER XFlash 5060FQ Annual EDS detector. The images taken were then analyzed and annotated with the Quartz PCI Version 9 (Quartz Imaging Corporation). The detailed puller parameters are as follows: heat – 580, pull – 20, velocity – 22, time – 250, pressure – 620, and ramp – 583. After each pull, the jaw temperature was allowed to cool down to 35 to 40 °C to ensure reproducibility. (Note: The wear and tear state of the filament box has a significant impact on the final shape of the emitter, and continuous adjustment of the puller parameters is necessary to ensure reproducibility.)

**C. Details of the MS parameters used for native top-down and complex-up analysis**

**Supplementary information Table 1: Parameter summary for top-down and complex-up analysis performed in this study.** The key MS2 parameters for nTD and complex-up experiments performed from *in vitro* liposome and *E. Coli* membrane vesicles are shown. 6+, 7+, and 19+ charge states of the monomer, dimer, and tetramer of VAMP2, semiSWEET, and AqpZ, respectively, were isolated and then fragmented with the EChcD approach. Similarly, 25+, 34+, and 19+ charge states of DLDH dimer, BAM heteropentamer, and BAMA-E heterodimer were isolated respectively, and then subjected to complex up analysis via HCD or top-down analysis via EChcD.

|  | | | | | **ExD cell parameters** | | | | | | | | | | | | | | |
| --- | --- | --- | --- | --- | --- | --- | --- | --- | --- | --- | --- | --- | --- | --- | --- | --- | --- | --- | --- |
|  | **precursor (oligomeric state)** | **Experiment type** | **resolution (@400 m/z)** | **HCD energy (V)** | **L1** | **L2** | | **LM3** | | **L4** | | **FB** | | | **LM5** | | **L6** | | **L7** |
| in vitro liposome | VAMP2 6+ | top-down | 100,000 | 80 | 1 | -40 | | 1 | | 3 | | -3 | | | 1 | | -40 | | 1 |
|  | semiSWEET 7+ (dimer) | top-down | 100,000 | 170 | 1 | -35 | | 0 | | 6 | | 0 | | | 0 | | -35 | | 1 |
|  | AqpZ 19+ (tetramer) | top down | 200,000 | 250 | 1 | -30 | | 4 | | 1 | | -5 | | | 4 | | -30 | | 1 |
| *E. Coli* membrane vesicle | DLDH 25+ (dimer) | top-down | 100,000 | 250 | 1 | -30 | | 5 | | 6.5 | | -0.5 | | | 5 | | -30 | | 1 |
|  | DLDH 25+ (dimer) | complex up | 12,500 | 80 | N/A | | | | | | | | | | | | | | |
|  | DLDH 17+ (monomer) | top-down | 100,000 | 150 | N/A | | | | | | | | | | | | | | |
|  | RBSB 17+ (monomer) | top-down | 100,000 | 140 | N/A | | | | | | | | | | | | | | |
|  | Superoxide dismutase  (Monomer) | top-down | 100,000 | 175 | N/A | | | | | | | | | | | | | | |
|  | Tryptophanase | top-down | 100,000 | 240 | 1 | | -35 | | 3 | | 5.5 | | -0.5 | 3 | | -35 | | 1 | |
|  | BAM 34+ (heteropentamer) | complex up | 6,250 | 225 | N/A | | | | | | | | | | | | | | |
|  | BAM 19+ (heterodimer, subunit A&E) | top-down | 100,000 | 285 | 0 | -30 | | 7.3 | | 5 | | 2 | | | 7.3 | | -30 | | 0 |

**D. Details of the software employed and specific parameters used for all the MS analyses**

**Supplementary Information Table 2:** Summary of the software used for data analysis and visualization.

| **PROCESS** | **SOFTWARE USED** | **RATIONALE FOR USE** |
| --- | --- | --- |
| MS2 Data Deconvolution | FlashDeconv, ExDViewer, and YADA3 | FlashDeconv, ExDViewer, and YADA3 were used to deconvolute the top-down MS/MS data. Both of these software were used to cross-validate the deconvoluted results among themselves and better identify falsely deconvoluted masses. |
| MS2 Data Deconvolution | UniDec | UniDec was used to deconvolute MS1 data of intact protein complexes to determine the masses of the protein complexes present. |
| Protein ID | ProSight Native and ProsightLite | ProsightNative was used to identify the intact protein complexes through complex-up and top-down analysis using database search. ProsightLite was used for targeted search. Prosight, native or Lite-mode, was used to determine the statistical validity of the matched sequence coverage. |
| Data visualization | FreeStyle and ExD viewer | Freestyle, the flagship software from ThermoFisher, was used for all MS and MS/MS data visualization. ExD viewer was used to visualize the annotation of fragment ions since, in our experience, it provided a more customizable graphical user interface while visualizing matched top-down data to a sequence. |
| Figure Generation | Adobe Illustrator | Adobe Illustrator was used to generate the figures |

**Parameters used for top-down data analysis:**

ProsightNative and ProsightLite were used to identify proteins from the top-down MS/MS data and validate the sequence match. While using ProsightNative, we used the established step-wise process while determining the IDs of multimeric complexes[^7^](https://sciwheel.com/work/citation?ids=16989075&pre=&suf=&sa=0&dbf=0). In short, for multimeric complexes, the protocol of using ProsightNative starts with determining the precursor mass of the overall complex from the MS1 native spectra, followed by mass annotations of the individual masses of the monomer formed in the complex-up data. For these deconvolutions, the in-built UniDec[^8^](https://sciwheel.com/work/citation?ids=1484201&pre=&suf=&sa=0&dbf=0) module was used to deconvolute and determine the masses of the individual complexes and ejected subunits. These individual monomeric masses are then saved as possible precursor masses while analyzing the top-down data of the overall complex. Post-top-down ID, these individual masses are also used to stitch together to determine the stoichiometry of the detected complex. In case all subunits are not detected as monomers in the complex-up data, the missing monomeric masses can be manually entered into ProsightNative. For the top-down ID, ProsightNative was first used to determine the ID of the fragmented proteins and determine. The data were searched against the *E. coli* database downloaded from UniProt. In the case of identifying VAMP2, semiSWEET, and AqpZ, the expressed sequences were manually added to the database in the FASTA format. While doing the database search, a 10ppm tolerance was used while matching the fragment ions. For all EChcD data, both b/y and c/z type ions were used, along with standard water and ammonia loss. For the HCD-only data, only b/y ion types were used. In both cases, ProsightNative uses the complex-up experiment-derived monomeric masses as possible precursor masses. Typically, a 10Da cut-off was used here. Matched protein IDs and their P-score were further validated by targeted analysis using ProsightLite[^9^](https://sciwheel.com/work/citation?ids=15190241&pre=&suf=&sa=0&dbf=0). Since deconvolution plays an important role in the top-down data analysis, we also independently deconvoluted the same data using both FlashDeconv[^10^](https://sciwheel.com/work/citation?ids=9929122&pre=&suf=&sa=0&dbf=0) the YADA3 algorithm[^11^](https://sciwheel.com/work/citation?ids=17793121&pre=&suf=&sa=0&dbf=0), as well as the inbuilt deconvolution algorithm of ExDViewer. The deconvoluted data, in mzml format, was fed into ProsightLite to validate the matched ions and determine the P-score. Further, to remain completely independent from the Prosght, we also separately used ExDViewer, with its in the built deconvolution algorithm, to validate the matched ions.

**SUPPLEMENTARY METHODS REFERENCES**

[1.    Panda, A. *et al.* Direct determination of oligomeric organization of integral membrane proteins and lipids from intact customizable bilayer. *Nat. Methods* **20**, 891–897 (2023).](https://sciwheel.com/work/bibliography/14760934)

[2.    Weber, T. *et al.* SNAREpins: minimal machinery for membrane fusion. *Cell* **92**, 759–772 (1998).](https://sciwheel.com/work/bibliography/53203)

[3.    Mahal, L. K., Sequeira, S. M., Gureasko, J. M. & Söllner, T. H. Calcium-independent stimulation of membrane fusion and SNAREpin formation by synaptotagmin I. *J. Cell Biol.* **158**, 273–282 (2002).](https://sciwheel.com/work/bibliography/53224)

[4.    Krishnakumar, S. S.](https://sciwheel.com/work/bibliography/235735) *[et al.](https://sciwheel.com/work/bibliography/235735)* [Conformational dynamics of calcium-triggered activation of fusion by synaptotagmin.](https://sciwheel.com/work/bibliography/235735) *[Biophys. J.](https://sciwheel.com/work/bibliography/235735)***[105](https://sciwheel.com/work/bibliography/235735)**[, 2507–2516 (2013).](https://sciwheel.com/work/bibliography/235735)

[5.    Laganowsky, A. *et al.* Membrane proteins bind lipids selectively to modulate their structure and function. *Nature* **510**, 172–175 (2014).](https://sciwheel.com/work/bibliography/464418)

[6.    Gupta, K. *et al.* The role of interfacial lipids in stabilizing membrane protein oligomers. *Nature* **541**, 421–424 (2017).](https://sciwheel.com/work/bibliography/2992956)

[7.    Durbin, K. R. *et al.* ProSight Native: Defining Protein Complex Composition from Native Top-Down Mass Spectrometry Data. *J. Proteome Res.* **22**, 2660–2668 (2023).](https://sciwheel.com/work/bibliography/16989075)

[8.    Marty, M. T.](https://sciwheel.com/work/bibliography/1484201) *[et al.](https://sciwheel.com/work/bibliography/1484201)* [Bayesian deconvolution of mass and ion mobility spectra: from binary interactions to polydisperse ensembles.](https://sciwheel.com/work/bibliography/1484201) *[Anal. Chem.](https://sciwheel.com/work/bibliography/1484201)***[87](https://sciwheel.com/work/bibliography/1484201)**[, 4370–4376 (2015).](https://sciwheel.com/work/bibliography/1484201)

[9.    Fellers, R. T. *et al.* ProSight Lite: graphical software to analyze top-down mass spectrometry data. *Proteomics* **15**, 1235–1238 (2015).](https://sciwheel.com/work/bibliography/15190241)

[10.   Jeong, K. *et al.* FLASHDeconv: Ultrafast, High-Quality Feature Deconvolution for Top-Down Proteomics. *Cell Syst.* **10**, 213-218.e6 (2020).](https://sciwheel.com/work/bibliography/9929122)

[11.   Yada 3.0. http://patternlabforproteomics.org/yada3/#_Toc108263859.](https://sciwheel.com/work/bibliography/17793121)
